# Presymptomatic pharmacological inhibition of mGluR5 improves survival in a mouse model of prion diseases

**DOI:** 10.1186/s40478-026-02235-9

**Published:** 2026-01-29

**Authors:** Yue Wang, Behnam Mohammadi, Christiane Hartmann, Kristin Hartmann, Edda Thies, Andreu Matamoros-Angles, Cheng Fang, David A. Harris, Jörg Tatzelt, Hermann C. Altmeppen, Diego Sepulveda-Falla, Stephen M. Strittmatter, Markus Glatzel, Susanne Krasemann

**Affiliations:** 1 Institute of Neuropathology, University Medical Center Hamburg-Eppendorf, Hamburg, Germany; 2 Department of Biochemistry and Cell Biology, Boston University/Chobanian & Avedisian School of Medicine, Boston, MA USA; 3 Department of Biochemistry of Neurodegenerative Diseases, Institute of Biochemistry and Pathobiochemistry, Ruhr University Bochum, Bochum, Germany; 4 Cluster of Excellence RESOLV, Ruhr University Bochum, Bochum, Germany; 5 Departments of Neuroscience and Neurology, Yale School of Medicine, New Haven, USA; 6 Present Address: Klinik und Poliklinik für Neurologie, Universitätsmedizin Rostock, Gehlsheimer Straße 20, 18147 Rostock, Germany; 7 Present Address: Faculty of Medicine University of Vic‐Central University of Catalonia (UVic‐UCC), 08500 Vic, Barcelona Spain; 8 Annovis Bio, 101 Lindenwood Dr., PA 19355 Malvern, USA

**Keywords:** Alzheimer’s disease, Metabotropic glutamate receptor 5, Neurodegeneration, Prion protein, CTEP, Silent allosteric modulator

## Abstract

Toxic signaling of oligomeric protein species via binding to the cellular prion protein (PrP^C^) is implicated in various neurodegenerative diseases, including Alzheimer’s (AD) and Parkinson’s disease (PD). Metabotropic glutamate receptor 5 (mGluR5) has been identified as a PrP^C^ signaling partner, and pharmacological inhibition of mGluR5 was shown to improve cognitive performance and rescue long-term-potentiation (LTP) impairment in both in vitro and in vivo models of AD and PD. Prion diseases are another group of fatal neurodegenerative disorders that are characterized by templated misfolding of endogenous PrP^C^ itself into the disease-driving counterpart PrP^Sc^. Besides its role in the self-propagating misfolding cascade and aggregation, PrP^Sc^ also acts as a toxic PrP^C^ ligand in aberrant signaling through mGluR5. Therefore, targeting metabotropic glutamate receptors has been proposed as a therapeutic strategy for the intervention of prion disease. In this study, we investigated the impact of long-term oral administration of two different selective mGluR5 inhibitors, the negative allosteric inhibitor CTEP and a *Silent Allosteric* mGluR5 *Modulator* (SAM), in a mouse model of prion disease. Our findings demonstrate that treatment initiated during the preclinical phase significantly prolonged the survival of mice, whereas treatment starting after the onset of symptoms was no longer effective. Early treatment also delayed the formation of spongiosis, a pathological hallmark of prion diseases, but did not alter PrP^Res^ levels. Preclinical dysregulation of mGluR5 could be shown in the mouse and a non-human primate model for prion diseases. Interestingly, in primary neurons, subacute treatment with CTEP blocked Aβ-induced, but not PrP^Sc^-associated synaptotoxicity. Thus, modes of action might differ markedly from those observed in models of Alzheimer’s disease. Together, although our data show that targeting mGluR5 may be an efficient therapy, however, since treatment needs to be started early during prion disease progression, the narrow therapeutic window limits its therapeutic application in human prion diseases.

**Supplementary Information:**

The online version contains supplementary material available at 10.1186/s40478-026-02235-9.

## Introduction

Despite being rather rare, prion diseases can be regarded as the prototype of cerebral proteinopathies, characterized by a cascade in which the cellular prion protein (PrP^C^) undergoes conformational conversion into its pathogenic isoform, PrP^Sc^ [1, 10, 48]. This misfolding process leads to the formation of oligomeric and fibrillar aggregates and ultimately results in progressive neuronal degeneration [48]. PrP^Sc^, the disease-associated protein isoform, plays a key role in templating the progressive misfolding of native PrP^C^, promoting aggregation, and facilitating the formation of transmissible units, termed prions, thereby contributing to disease progression. Beyond serving as a substrate for PrP^Sc^ propagation, PrP^C^ has also been identified as a common receptor for various misfolded β-sheet-rich oligomeric protein species, including amyloid-β (Aβ), phospho-tau, α-synuclein and PrP^Sc^, leading to induction of synapto- and neurotoxic signaling processes [11, 12, 15, 17, 33, 49, 50, 55]. Since PrP^C^ is anchored to the outer leaflet of the plasma membrane, intracellular signaling requires additional co-receptors or transmembrane binding partners. Among these, the metabotropic glutamate receptor 5 (mGluR5) has emerged as a critical mediator of Aβ-induced synaptotoxicity, particularly in Alzheimer’s disease (AD) models [7, 19, 24, 58].

Genetic and pharmacological inhibition of mGluR5 ameliorate cognitive decline and reduce plaque formation in mouse models for AD-associated amyloidosis [20, 22, 54]. In a mouse model of prion disease, inhibition of group-I metabotropic glutamate receptors over the entire experimental course delayed disease progression and improved motor function [18]. Moreover, pharmacological inhibition of mGluR5 rescued the impairment of neuronal LTP caused by oligomeric α-synuclein in primary neurons [15]. CTEP (2-chloro-4-((2,5-dimethyl-1-(4-(trifluoromethoxy)phenyl)-1*H*-imidazol-4-yl)etynyl)pyridine) is a highly selective, brain-penetrant, orally bioavailable negative allosteric modulator (NAM) of mGluR5, exhibiting high affinity and specificity to mouse, human and rat receptors. Compared to earlier mGluR5 antagonists such as 2-methyl-6-(phenylethynyl)pyridine (MPEP), CTEP offers improved pharmacokinetics and sustained receptor occupancy [36]. However, complete inhibition of mGluR5 may result in adverse side effects. Studies in a rat model revealed cognitive deficits following mGluR5 inhibition with MPEP [8].

To overcome these limitations, a silent allosteric modulator (SAM, BMS-984923, ALX-001) of mGluR5 has been developed. This mGluR5 modulator selectively interferes with the pathological interaction of PrP^C^ bound to oligomeric protein species e.g. the Aβo/PrP^C^/mGluR5 signaling axis, while preserving physiological glutamatergic signaling [20]. SAM has shown promise in mitigating AD-related phenotypes in preclinical models [20, 54].

Given the dual role of PrP^C^ in prion diseases, both as a substrate for continuous PrP^Sc^ formation and as a cell surface receptor for mediating toxic oligomer-induced signaling, we aimed to explore the inhibition of mGluR5 signaling as a therapeutic approach. To distinguish potential PrP^Sc^-induced neurotoxic from physiological functions of mGluR5 during prion disease progression, we investigated whether chronic oral treatment with either the negative allosteric inhibitor CTEP or the silent allosteric mGluR5 modulator [20] could improve survival and influence disease progression in a prion disease mouse model. We found that preclinical long-term treatment with each mGluR5 inhibitor significantly prolonged survival and reduced spongiosis, yet without affecting brain PrP^Res^ levels. In cognitive testing, CTEP improved function in the prion-infected mice. mGluR5 was shown to be dysregulated in the hippocampus already during the preclinical phase in the mouse model, and also in a non-human primate model for prion diseases [28]. In contrast, treatment with either drug initiated after the onset of symptoms had no measurable impact on disease progression. Our findings, despite showing efficacy against prion disease, highlight a narrow time window for mGluR5-targeted therapeutic interventions in prion diseases, underscoring differences in the therapeutic response compared to mouse models of AD-associated pathology.

## Materials and methods

### Ethics statement

The animal experiments were in strict accordance with the principles of laboratory animal care (NIH publication No. 86 − 23, revised 1985) and the recommendations in the Guide for the Care and Use of Laboratory Animals of the German Animal Welfare Act on protection of animals. The experiments comply with current laws of Germany and the protocol for the work with mice was approved by the Ethical Committee of the *Freie und Hansestadt Hamburg*, *Amt für Gesundheit und Verbraucherschutz* (Permit numbers: V 1300/591 − 00.33 and V1305/591 − 00.33). All inoculations of mice were performed under ketamine hydrochloride and xylazine hydrochloride anaesthesia, and all efforts were made to minimize suffering, including application of intraoperative analgesics.

All procedures involving primates were performed in accordance with European and German legal and ethical regulations (which are in line with the recommended practices of the use of non-human primates in research) and approved by the responsible boards and authorities. All procedures involving the rhesus macaques described in this manuscript were started in 2002 and finalized in 2006 at the German Primate Center (Göttingen, Germany) in accordance with the German Animal Welfare Act and the Council Directive 86/609/EEC (Permit 33.42502/08–08.02 LAVES, Lower Saxony, Germany).

### Non-human primates

Infection studies of rhesus macaques with human vCJD prions between 2002 and 2006 were already described [28], and archived formalin-fixed paraffin-embedded brain tissue samples were used for this work. Briefly, captive-bred, primate pathogen-free rhesus macaques (*Macaca mulatta*) were inoculated intraperitoneal in 2002 with 10 mL of a 10% (w/v) homogenate in PBS of human vCJD-affected brain (prion protein gene codon 129 MM, PrP^Sc^ type 4 according to Hill et al. [23], kindly provided by John Collinge, London). The control animal received saline only. Some animals had to be sacrificed preclinically due to intercurrent illness. One animal progressed to clinical prion disease in 2006 and was sacrificed 4 weeks after onset of first clinical symptoms (slowness, weight loss, trembling). Brain tissues were fixed in 4% formalin, inactivated in formic acid, post-fixed, and embedded in paraffin for immunohistochemical analyses.

### Mice

Mice were purchased from Charles River (Germany) and were maintained under pathogen-free conditions. Eight weeks old male and female C57BL6 mice (for CTEP treatment) or female C57BL/6 mice (for SAM treatment) were intracerebrally inoculated with a 10% brain homogenate of Rocky Mountain Laboratory (RML) strain 5.0 prions [47] (3 × 10^5^ logLD_50_ (high dose) for CTEP experiments and 1 × 10^5^ logLD_50_ (high dose) for SAM experiments). Control animals received mock homogenate (i.e., brain homogenate from uninfected CD-1 mice). Mice were anesthetized with ketamine/xylazine hydrochloride prior to inoculation and received intraoperative analgesics to improve postoperative recovery. A subset of mice was sacrificed at preclinical day 90 (after 30 days of CTEP treatment starting at day 60 after prion inoculation). Moreover, a subset of mice was sacrificed on an early clinical time point at day 115 post prion injection (dpi) (for SAM treatment starting at both, 60 and 90 dpi). To determine the incubation time of prion disease, each experimental cohort was allowed to progress to terminal prion disease (defined by presenting with slowness, ataxia, weight loss, trembling and ungroomed fur). One brain hemisphere was collected and processed for immunohistochemistry while the other was either dissected into different brain regions or kept intact and stored at -80 °C for biochemical analyses. Control mice were sacrificed at corresponding time points for analysis.

### Chemicals

If not otherwise indicated, all chemicals were purchased from Sigma-Aldrich (USA).

### Drug treatment

The highly selective mGluR5 antagonist CTEP was purchased at MCE (MedChem Express, USA) and applied via a standard rodent diet. Based on published work, where CTEP was shown to achieve uninterrupted mGluR5 occupancy with 2 mg/kg p.o. per 48 h in mice [36]. Thus, here, the mGluR5 inhibitor CTEP was mixed into a standard rodent diet at 4 mg of drug per kilogram of chow (Altromin, Germany). Food pellets contained CTEP at 20 µg per 5 g of diet to achieve this dose per mouse (average weight of 20 g), respectively, corresponding to a dose of approximately 2 mg/kg per mouse in 48 h. Control chow did not contain CTEP but was otherwise prepared identically.

SAM compound (BMS-984923, ALX-001) was incorporated into OpenStandard Diet (#D11112201i, Research Diets, Inc., USA) at 50 mg SAM per kg food (250 µg per 5 g of chow); control chow was the same purified diet without the compound. The dosage of the drug in the food was calculated taking into account the average amount of food eaten by a mouse, and adjusted to be equivalent to ingesting 7.5 mg/kg per day [54].

Oral bioavailability and efficient brain tissue exposure and receptor occupancy for both drugs have been tested in different experimental animal models and confirmed before (see summary for CTEP: https://file.medchemexpress.com/batch_PDF/HY-15445/CTEP-DataSheet-MedChemExpress.pdf and [36, 54]. Both drugs are highly selective for mGluR5 and effective in nanomolar quantities. The IC_50_ of CTEP is 2.2 nM [36]. SAM has a competitive antagonism of MPEP binding at K_i_ of 0.6 nM, but no detectable agonist and antagonist activity at mGluR5 signalling [20].

### Assessment of nest building behaviour

Nest building was closely monitored and staged every ten days from day 60 until the end of the experiment. Mice were housed in groups of three mice per cage. Nest building quality was assessed using a four-stage scoring system, with evaluations performed blinded with regard to the treatment groups. Assessment was conducted 24 h after fresh paper nesting material was provided to each cage: 3 = proper nest building (all paper used and mice could hide completely in their nest, paper is tightly packed to form a nestlet); 2 = disorganized nest (paper not properly arranged and only loosely packed, not all paper is used to build the nestlet, mice are not able to completely hide in nest anymore); 1 = flat paper on the ground (paper is still used as a place for sleeping); 0 = (flat) paper is scattered all over on the ground and soaked wet with mouse pee (mice do not use it as a nest anymore, this score/time-point defined the termination of the experiment and the mice were sacrificed).

### Western blot analysis

For western blot analyses, brains were homogenised (FastPrep FP120, Qbiogene, France) at 10% (w/v) in RIPA buffer (150 mM NaCl, 1% NP-40, 0.5% DOC, 0.1% SDS, 50 mM Tris-HCl pH 8.0) and a subset of samples was digested with proteinase K (PK) (20 µg/mL) (Roche, Germany) for 1 h at 37 °C. Digestion was stopped by addition of 10× sample buffer and boiling for 10 min. Samples were analyzed by SDS-page (AnykD, Biorad, USA), transferred to nitrocellulose membranes (0.2 μm pore size, BioRad) at 400 mA for 1 h. Total protein staining was performed according to the manufacturer’s protocol (Revert™ Total Protein Stain kit; Licor). Thereafter, membranes were blocked for 1 h at room temperature (RT) in 5% milk powder in TBS-T buffer and incubated overnight at 4 °C with anti-PrP antibody POM1 (Millipore), POM2 (Millipore), Actin (Sigma-Aldrich) or mGluR5 (1:1,000; #55920 Cell Signaling). After incubation for 1 h at RT with an HRP-conjugated anti-mouse or anti-rabbit secondary antibody (1:5,000 in blocking buffer), signals were detected with either Pierce ECL Pico or SuperSignal West Femto substrate (Thermo Scientific) and visualized and densitometrically quantified with a ChemiDoc imaging station and VersaDoc (both from BioRad). Total Western blot images for all blots are displayed in the Supplementary Figs. S7 and S8.

### Immunohistochemistry

All tissues, including control tissues, were fixed with 4% buffered formalin, prion infectivity was inactivated by immersion in 98% formic acid for one hour (mouse brains) or two hours (tissue samples of non-human primates), tissue samples were post-fixed in 4% buffered formalin overnight, and processed for paraffin embedding. Sections (3 μm thick) were subjected to Hematoxylin and Eosin (H&E) staining according to standard procedures. For detection of glial fibrillary acidic protein (GFAP, Dako M0761), ionized calcium binding adaptor molecule 1 (IBA1, Wako Pure Chemical Industries 019-19741) in mouse brain tissue, or mGluR5 (1:200; #55920 Cell Signaling) in mouse and non-human primate brain tissue by immunohistochemical staining, a Ventana Benchmark XT autostainer was used (Ventana, Tuscon, Arizona, USA). Deparaffinization and antigen retrieval of brain tissue sections was performed, followed by incubation with primary antibody for 1 h. Anti-rabbit or anti-mouse Histofine Simple Stain MAX PO Universal immunoperoxidase polymer (Nichirei Biosciences, Wedel, Germany) were used as secondary antibodies. Detection of secondary antibodies and counterstaining was performed with an ultraview universal DAB detection kit from Ventana. Data acquisition was performed using a Leica DMD108 digital microscope.

The degree of tissue spongiosis was assessed in a semi-quantitative manner. Regions of interest within each hemisphere (cortex, hippocampus, thalamus, and cerebellum) were examined under a light microscope, and scored for vacuolar lesions on a scale of 0–4 as follows: 0 (i.e., no or rare vacuoles), 1 (i.e., a moderate number of small vacuoles), 2 (i.e., numerous small vacuoles with sparsely scattered large vacuoles), 3 (i.e., a large number of vacuoles of different sizes, evenly distributed), and 4 (i.e., numerous vacuoles with fusion among them). Vacuole size comparisons were made based on the average size of granule cell nuclei in the dentate gyrus as reference, and were performed only within the same region. At least three mice per group were used, with at least two consecutive sagittal sections per mouse.

PrP^Sc^ detection was performed as described before [29]. Briefly, mounted tissue sections (3 μm thick) were pretreated with 98% formic acid for 30 min, and autoclaved in citrate buffer (pH 6,0) for 5 min at 121 °C. Further processing was performed on an automated staining machine (BenchMarkTX). Sections were digested with low concentration of PK (Ventana protease) for 16 min, incubated in Superblock for 10 min and then incubated with the PrP-specific antibody Saf 84 for mouse tissue (1:100; Cayman Chemical, USA) or 3F4 for non-human primate tissue (1:100; #MAB1562 Merck, Germany), followed by detection with an HRP-coupled secondary antibody.

Positive signal area was quantified in three to four different sections each for PrP^Sc^ using the Analyze function in the ImageJ 1.52e software [51]. Total area for each section was 273,000 µm^2^.

### Immunofluorescence staining

For immunofluorescence staining, brain paraffin sections were cut at 3 μm thickness and thoroughly deparaffinized by 2 × 20 min incubation in Xylene and a descending alcohol row. Antigen retrieval was performed by pressure boiling the sections in Universal R buffer (#AP0530-500 with 2100 Antigen Retriever; Aptum, UK) for 20 min. Sections were briefly rinsed and blocked for 1 h (MAXblock, Active Motif). Antibodies against mGluR5 (1:200; #55920 Cell Signaling), IBA1 (1:200; #234308 Synaptic Systems or 1:500; #019-19741 Wako), or P2RY12 (1:5,000; #476011 Synaptic Systems) were incubated overnight at 4 °C. After intensive washing, AlexaFluor488- and AlexaFluor555-coupled secondary antibodies were applied for 1.5 h. Sections were washed again, counterstained with DAPI and mounted in Fluoromount-G (SouthernBiotech, USA). Data acquisition was performed using a Leica Sp5 confocal microscope and Leica application suite software (LAS-AF-lite) or ECHO Revolve microscope (ECHO, USA). Figures were assembled using Adobe Photoshop 2021. For the quantification of mGluR5 signal, regions of interest (ROIs) measuring 300 μm × 300 μm, from the stratum radiatum of the CA1 field in Ammon’s horn (CA) of the hippocampus, were randomly selected from 20× objective images of each mouse using Fiji software. The mean grey intensity from three ROIs represented each mouse. Data was collected and analyzed using GraphPad Prism (GraphPad, USA).

### Expansion microscopy

Expansion microscopy staining on mouse brain paraffin sections was performed as previously described [43]. Briefly, sections were deparaffinized, underwent antigen retrieval, and were then anchored and embedded in the gel. Gelled sections were autoclaved, washed, and blocked, and mGluR5 was stained (1:100; #55920) together with MAP2 (1:200; #188004, Synaptic Systems) over 2 nights. After intensive washing, AlexaFluor 488 goat anti-rabbit and AlexaFluor 647 goat anti-guinea pig were used as secondary antibodies for detection of specific binding and sections were counterstained with DAPI. Gels were expanded in diluted PBS, and data acquisition was performed using a Leica Sp5 confocal microscope and Leica application suite software (LAS-AF-lite).

### Bulk RNA sequencing and data analysis

Total RNA was extracted from mouse hippocampus brain tissue using a tissue RNA Isolation Kit (#74104, RNeasy Kit, Qiagen) according to the manufacturer with one exception: To inactivate prion infectivity, tissues were homogenized in lysis buffer and incubated in it for 2 h at RT before further processing of the RNA. RNAs of all tissue samples showed an RNA integrity number (RIN) of > 8 and were processed for library preparation, which included enrichment of poly(A) selection of mRNA, fragmentation, and complementary DNA synthesis. Standard RNA sequencing (RNAseq) (next-generation sequencing) was performed at Genewiz (Azenta; Leipzig, Germany) on an Illumina NovaSeq platform with 2 × 150 bp paired-end reads configuration.

Raw reads were pre-processed by Cutadapt and Prinseq++. High-quality reads were aligned to the *Mus musculus* reference genome (GRCm39) obtained from GENCODE using STAR aligner. Mapped reads were counted using FeatureCounts. Principal Component Analysis (PCA) was conducted based on transcriptomic profiles to assess sample clustering and variability. Differentially expressed genes (DEGs) were obtained using the DESeq2 package in R with a false discovery rate (FDR, by Benjamini-Hochberg method) adjusted *p*-value ≤ 0.05. Functional enrichment analysis was performed using the String Database analytic functions with a similarity cutoff of 0.8 to reduce redundancy [57]. For data visualization, PCA and functional enrichment and volcano plots were created with the ggplot2 package [59].

### Primary neuronal cultures and measurement of synaptotoxicity

Subacute synaptotoxicity assays in low-density primary neuronal cultures were performed as previously described [13]. Briefly, an astrocyte feeder layer was generated. In parallel, hippocampal neurons from C57BL/6 mice were isolated from P0 pups. Neurons were seeded at 75 cells/mm^2^ on coverslips that were pre-treated with poly-L-lysine. After attachment, neurons on coverslips were inverted onto the astrocyte feeder layer. Neurons were kept in culture for 18–21 days prior to PrP^Sc^ or Aβ-derived diffusible ligand (ADDL) treatment, in the presence or absence of CTEP. After treatment for 24 h, neurons were fixed in 4% paraformaldehyde and stained with Alexa 488-phalloidin (ThermoFischer Scientific, MA) to visualize dendritic spines. Images were acquired on a confocal microscope (Zeiss 880) with a 63× objective. The number of dendritic spines was quantified using ImageJ software as described [13, 14].

PrP^Sc^ or ADDLs, used to treat primary neurons in the synaptotoxicity assay, were prepared as described before: see [13] for PrP^Sc^ and [5] for ADDLs.

### Statistical analysis

For statistical comparison of western blot data, Student’s *t*-test was used. For Kaplan-Meier survival curve calculation, the Log-rank (Mantel-Cox) test was applied, and for immunohistochemistry-based quantifications, a semi-quantitative measurement was used followed by group comparison with ANOVA, with levels for statistical significance set at *p*-values < 0.05 (*), < 0.01 (**) and < 0.001 (***). Statistical analysis was done in Prism (GraphPad; version 8).

## Results

### Chronic preclinical treatment with mGluR5 inhibitors improves survival in a prion disease mouse model

**Fig. 1:**
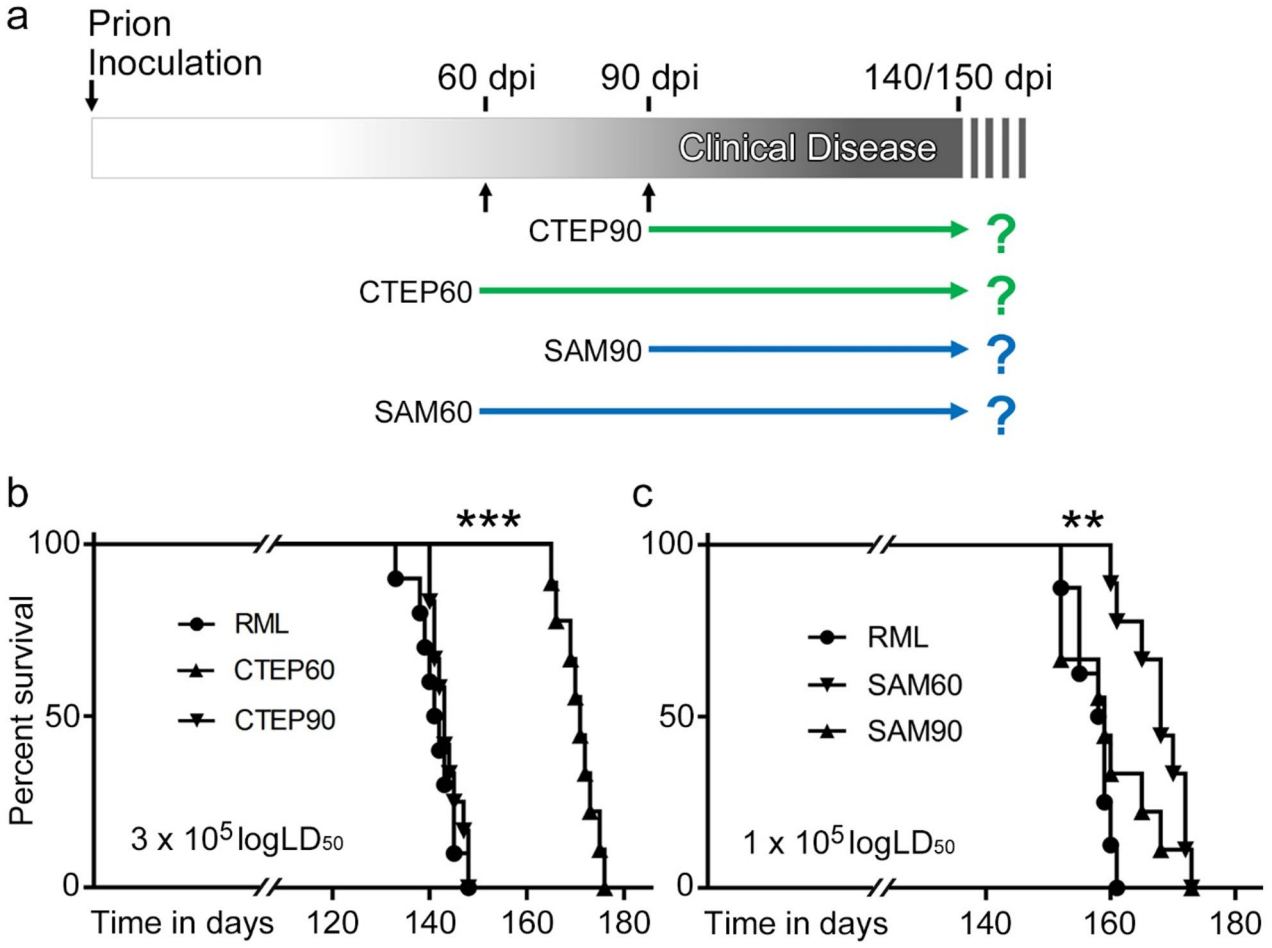
Oral preclinical treatment with mGluR5 inhibitors significantly prolong survival in a prion disease mouse model. **a** Schematic overview of the experimental outline including intracerebral inoculation with RML5.0 prions (at experimental day 0), oral CTEP and SAM treatments starting at 60- or 90-days post inoculation (dpi), and time points of analyses. The majority of animals were allowed to progress to terminal disease to assess survival kinetics. A subset of animals was analyzed at early clinical time points to determine the impact of oral treatment on prion-induced brain pathology. **b** Kaplan-Meier survival curve of prion-infected mice without (RML; *n* = 10) or with chronic oral CTEP treatment starting at 60 dpi (CTEP60; *n* = 9) or 90 dpi (CTEP90; *n* = 12). Female and male mice were both used in this experiment and distributed equally in the curves. No gender effect could be seen; ***p = 0.0002. **c** Kaplan-Meier survival curve of prion-infected mice without treatment (RML; *n* = 8) or with chronic oral SAM treatment from day 60 (SAM60; *n* = 9) or day 90 (SAM90; *n* = 9) post infection. Only female mice were used in this study; **p = 0.0051

To assess if pharmacological targeting of mGluR5 affects prion disease pathophysiology and progression, we infected 8 weeks old C57BL/6 mice with RML prions, a widely used, mouse-adapted prion strain. One group of mice each was chronically treated with either CTEP or SAM starting at 60 dpi (CTEP60; SAM60), which is considered the preclinical phase. The other groups started receiving CTEP or SAM beginning at 90 dpi (CTEP90; SAM90), when the first symptoms of prion disease such as a stiff tail appeared. Untreated prion-infected mice (RML) served as controls (see Fig. 1a for treatment scheme). Mock-inoculated mice served as negative controls or were fed with CTEP- or SAM-containing chow, respectively, to serve as treatment controls.

Mice were monitored until the onset of terminal disease and brain tissues were collected for biochemical and histological analyses. Prion-infected RML-only mice and CTEP90- and SAM90-treated groups exhibited similar disease durations of about 140–150 dpi (Fig. 1b, c). In contrast, mice treated with CTEP60- and SAM60 survived significantly longer (Fig. 1b, c). Interestingly, CTEP60 treatment, which results in complete inhibition of mGluR5, was superior to SAM60 treatment in extending survival in separate but comparable experiments. No gender-related differences in survival probability were observed in the CTEP60 group.

Before the onset of symptoms, nest building behavior was closely monitored every ten days from 60 dpi onwards to detect early behavioral changes. Loss of nest building ability is one of the first symptoms during the progression to clinical prion disease, for intracerebral inoculation with RML prions typically occurring around 90 dpi [9]. In line with survival data, no significant improvement in nest building could be detected in mice treated from 90 dpi onwards compared to untreated prion-infected controls (Supplementary Fig. S1). Treatment with SAM from day 60 onwards also did not improve nest building behavior (data not shown). However, treatment with CTEP from day 60 onwards significantly delayed the loss of nest building behavior (Supplementary Fig. S1).

### mGluR5 Inhibition does not influence PrP^Res^ levels but reduces spongiform lesions

**Fig. 2:**
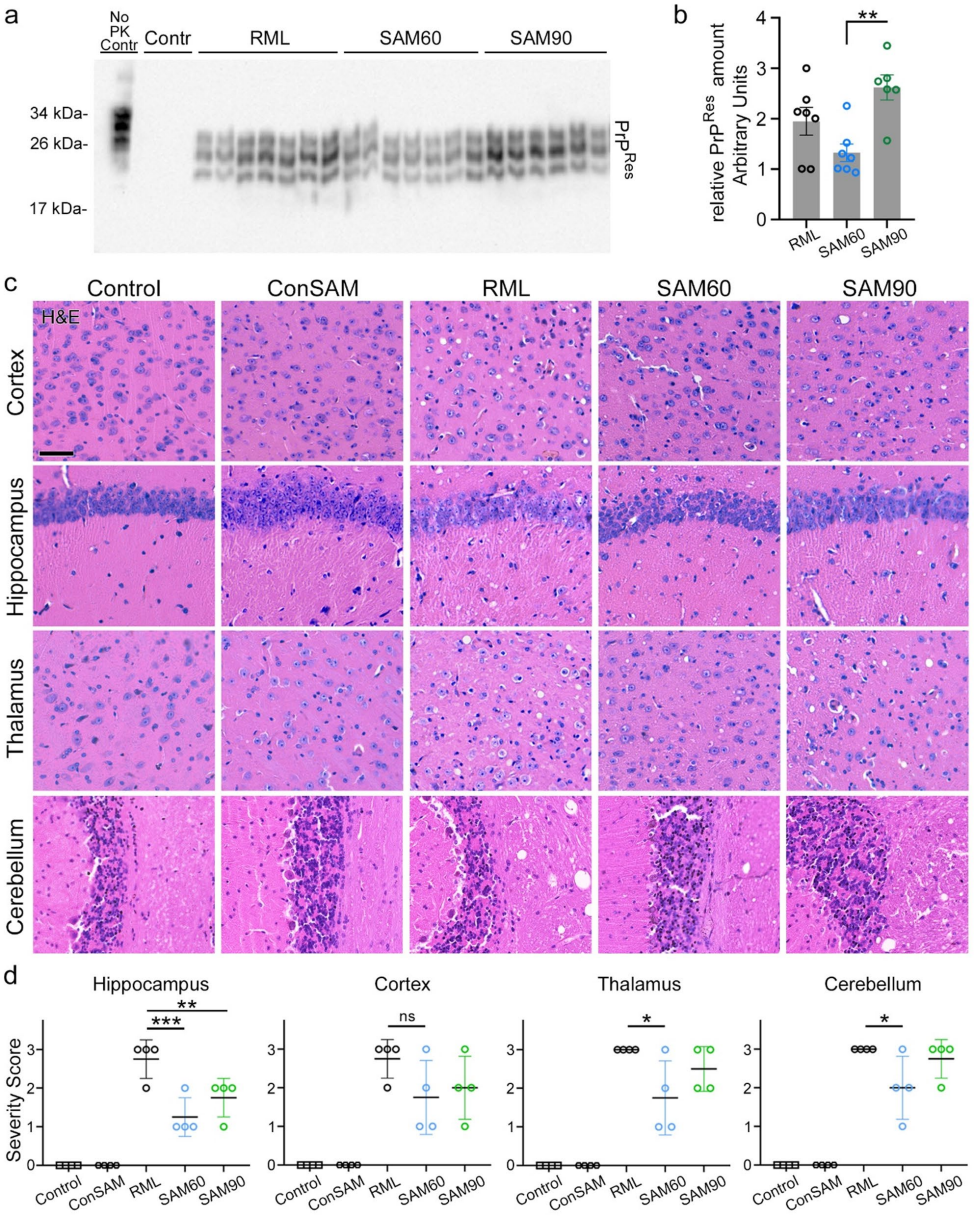
Preclinical treatment with SAM reduces levels of spongiosis, but not PrP^Res^levels, in the brain of prion-infected mice. **a** Representative western blot assessing PrP^Res^ levels after PK digestion from cortex tissue of SAM60, SAM90, RML, and control mice at 115 dpi (*n* = 6–7 per experimental group; *n* = 3 for healthy controls). **b** Densitometric quantification of PrP^Res^ levels from western blot. One-way ANOVA with Tukey’s multiple comparison test: **p = 0.0035). **c** Representative H&E staining of different brain areas at 115 dpi showing the degree of spongiform changes in RML-infected mice with and without SAM treatment, and in comparison to healthy controls. Scale bar: 50 μm. **d** Semi-quantitative assessment (by expert scoring) of spongiosis reveals a significant reduction of pathological changes only when SAM treatment started at 60 dpi (SAM60). One-way ANOVA with Dunnett’s multiple comparison test: Hippocampus ***p = 0.0002; **p = 0.0081; Thalamus *p = 0.0102; Cerebellum *p = 0.0163; ns = not significant

Since long-term intraperitoneal injections with CTEP has been shown to significantly reduce Aβ plaque formation and amounts of soluble Aβ oligomers in mouse models of AD-characteristic amyloidosis [22], we assessed whether SAM or CTEP would likewise influence deposition of PrP^Sc^ or levels of PrP^Res^ in our prion model. SAM60- and SAM90-treated mice, along with respective RML-positive and -negative controls were analyzed at 115 dpi via biochemical and immunohistochemical methods (Fig. 2). SAM treatment did not significantly alter levels of PrP^Res^ in prion-infected mice when compare to untreated RML mice when assessed by western blotting of brain tissue homogenates after proteinase K digestion (SAM60 *p* = 0.0774 in comparison to untreated prion-infected RML mice) (Fig. 2a, b). However, PrP^Res^ levels in the SAM60 group were significantly reduced when compared to the SAM90 group. Semi-quantitative analysis of spongiosis in H&E-stained brain sections showed a clear reduction of spongiosis upon SAM treatment, particularly in SAM60-treated mice (Fig. 2c, d). The SAM90 group showed a statistically significant reduction of pathology in the hippocampus and a similar non-significant trend in the cerebral cortex. In contrast, treatment had no detectable effect on microglia or astrocyte activation states (Supplementary Fig. S2).

**Fig. 3:**
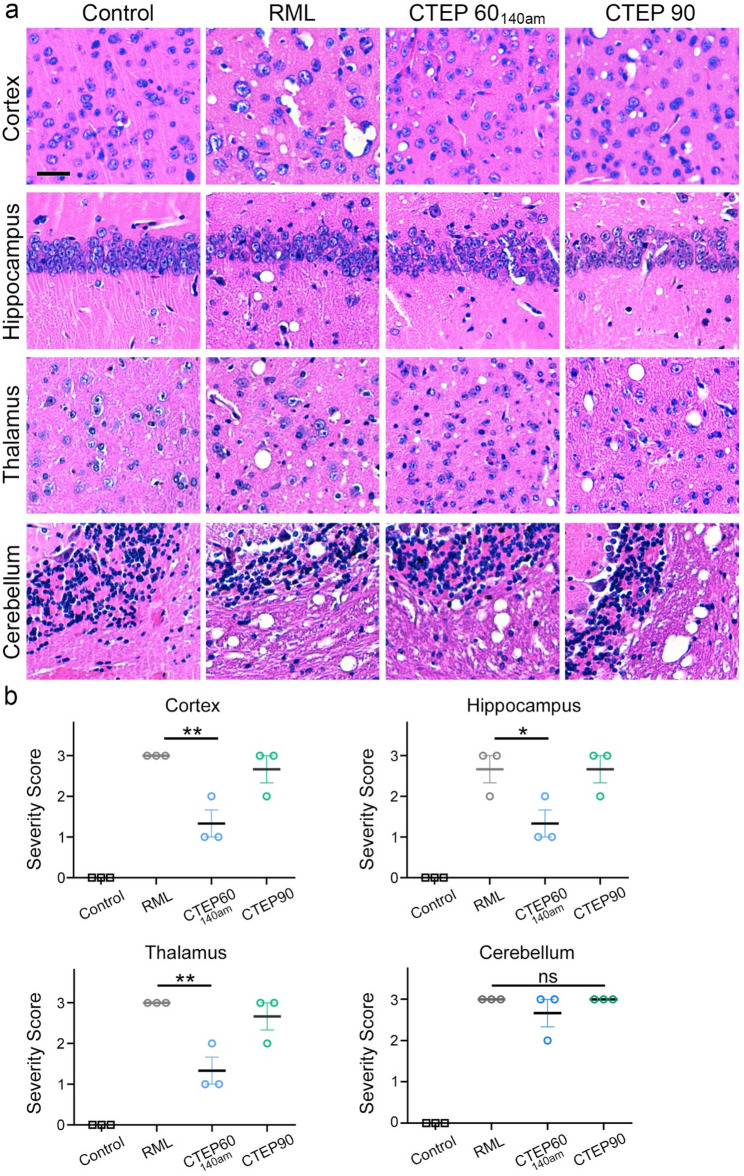
Preclinical treatment of prion-infected mice with CTEP reduces spongiosis. **a** Representative H&E staining of different brain regions in clinically diseased RML and CTEP90 mice, healthy control mice, and age-matched CTEP60_140_ mice (CTEP60_140am_) that were taken at the comparable time point at 140 dpi showing the degree of spongiform changes. Scale bar: 30 μm. **b** Semi-quantitative scoring shows a significant reduction in spongiosis only when CTEP treatment started at 60 dpi (CTEP60_140am_). One-way ANOVA with Dunnett’s multiple comparison test: Cortex **p = 0.0035; Hippocampus *p = 0.0321; Thalamus **p = 0.0035; Cerebellum = not significant

**Fig. 4:**
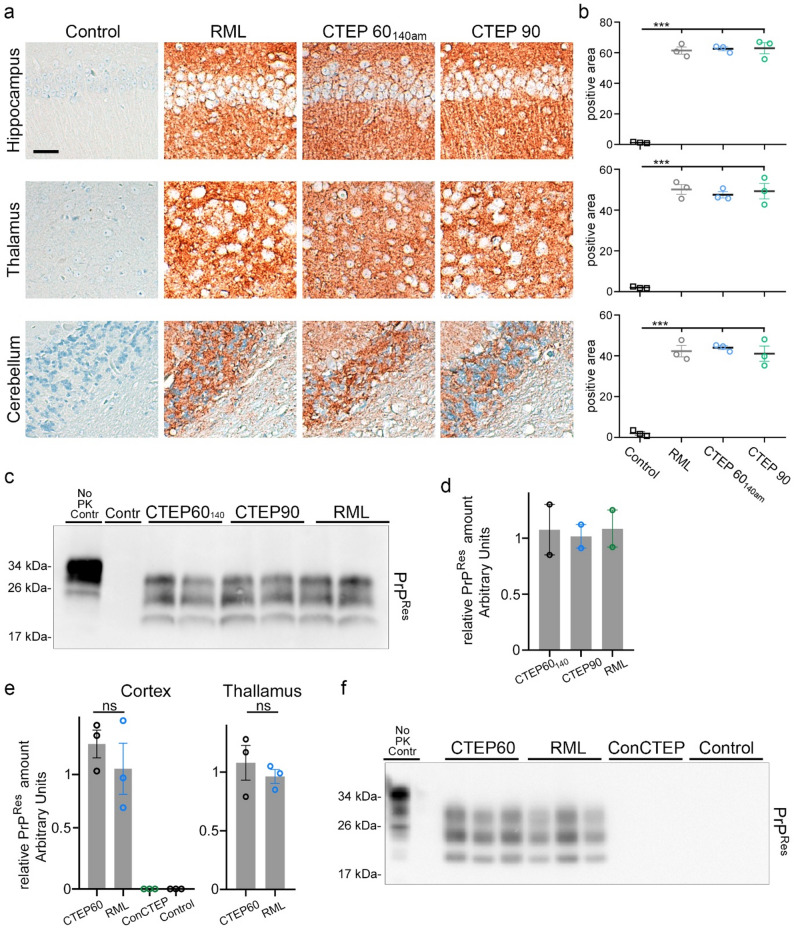
Preclinical treatment of prion-infected mice with CTEP does not change levels of PrP^Res^ or deposition of PrP^Sc^. **a** Representative IHC staining for deposition of PrP^Sc^ in different brain regions in clinically diseased mice and age-matched controls does not reveal major differences in PrP^Sc^ amounts and distribution pattern. Scale bar: 30 μm. **b** Quantification of PrP^Sc^ staining signal confirmed that treatment with CTEP did not change the formation of PrP^Sc^. **c** Representative western blot of PrP^Res^ after PK digestion at terminal and age-matched time points respectively, confirms that PrP^Res^ levels are unchanged by treatment. **d** Quantification of total signal of western blot was performed on a limited number of mice. **e** Quantification of total PrP^Res^ signal of western blot was performed at the early clinical day 90 after 30 days of treatment with CTEP in the mGluR5 abundant cortex and in the mGluR5 low-abundant thalamus. **f** Representative western blot of PrP^Res^ after PK digestion of cortex tissue show that CTEP treatment has no influence on PrP^Res^ levels

CTEP60 mice showed a significantly longer survival (171 ± 4 dpi) (Fig. 1b) compared to untreated prion-infected mice (140 ± 5 dpi). To allow better comparison of potential differences in brain pathology at a similar disease stage, a couple of CTEP60 animals (CTEP60_140am_) were sacrificed at 140 dpi, an age-matched (140am) time point to the clinical stage of CTEP90 (144 ± 3 dpi) and RML (140 ± 5 dpi) mice. To assess underlying neuropathological changes, brain tissues from RML, CTEP90, CTEP60_140am_, and control mice were analyzed by H&E staining for spongiosis and by immunohistochemical staining for abundance of astrogliosis (GFAP), microglia activation (IBA1), and PrP^Sc^ deposition in brain tissue sections (Fig. 3 and Supplementary Fig. S3). CTEP60_140am_ mice showed reduced spongiosis compared to the clinical stage RML and CTEP90 mice (Fig. 3a), with semi-quantitative assessment confirming significant reductions across all examined brain regions (Fig. 3b). There was no difference evident between RML and CTEP90 groups. As in the SAM-treated mice, we neither observed changes in the quantity, morphology, or signal intensity of astrocytes or activated microglia (Supplementary Fig. S3). We did not detect differences in PrP^Sc^ deposition amount or pattern in brain tissue sections when assessed by immunohistochemistry and quantification of positive signal across CTEP-treated groups when compared to untreated groups (Fig. 4a and b). Moreover, biochemical analyses of PrP^Res^ levels using western blots after proteinase K digestion on a limited number of mouse brain tissues confirmed these findings (Fig. 4c and d).

Our data showed that treatment with CTEP from day 60 significantly prolonged survival, while treatment from day 90 was ineffective. To better characterize this critical therapeutic window, we treated mice with CTEP from 60 dpi onwards and sacrificed already on day 90 (i.e., after 30 days of treatment). Mock-infected mice with and without CTEP treatment and prion-infected mice at 90 dpi served as control. Similar to the clinical time point, the biochemical analyses showed that PrP^Res^ levels also did not differ at this early time point in mice treated with CTEP compared to untreated mice (Fig. 4e and f). Interestingly, PrP^Res^ levels upon treatment with CTEP did neither differ in mGluR5 high-abundance brain regions (cortex; Fig. 4e and f) nor low-abundance brain areas (thalamus; Fig. 4e).

### Effect of mGluR5 Inhibition on transcriptomic signature is mild

**Fig. 5:**
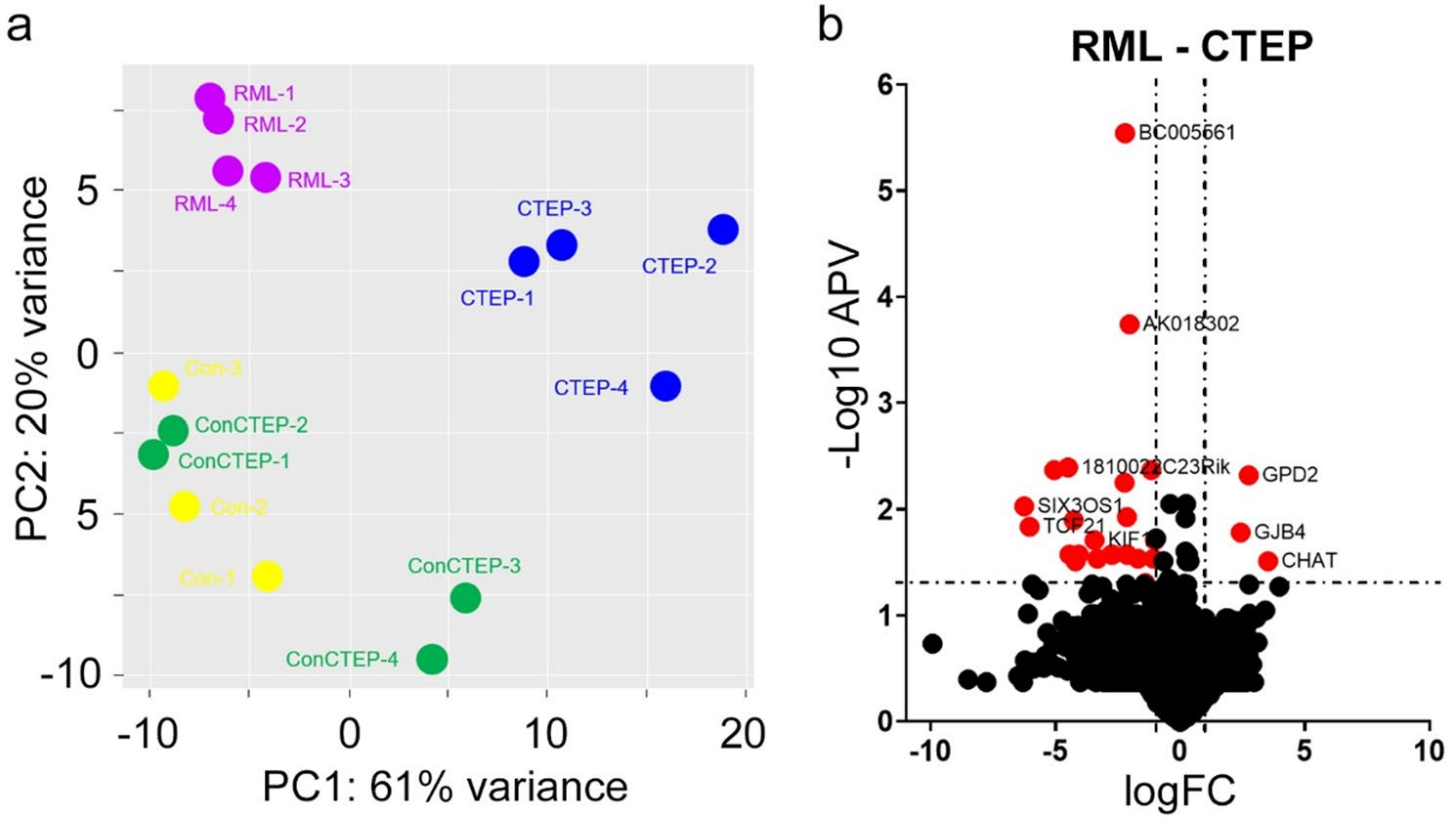
Preclinical treatment with CTEP alters mRNA expression profile in the brain of prion-infected mice. Transcriptome analysis of hippocampus tissue samples of mice at 90 dpi with or without prior treatment with CTEP for 30 days is shown. **a** Principal Component Analysis (PCA) plots showed sample distribution across groups: control mice (Con, *n* = 3), CTEP-treated control mice (ConCTEP, *n* = 4), prion-infected mice (RML, *n* = 4), CTEP-treated prion-infected mice (CTEP, *n* = 4). **b** Volcano plot of differentially expressed genes (DEGs) between prion-infected CTEP-treated mice (CTEP) and untreated prion-infected mice (RML), highlighted the top 28 significantly upregulated and downregulated genes (adj. *p* < 0.05, |log2FC| > 1)

To identify potential targets of the treatment, we then performed bulk RNA sequencing from hippocampal brain tissue from this preclinical time point. Interestingly, treatment of healthy mice with CTEP only led to very mild expression changes in the hippocampus, and PCA showed that healthy mice with and without CTEP treatment clustered together (Fig. 4a and Supplementary Fig. S4a). In contrast, PCA revealed that the hippocampal transcriptomic signature of CTEP-treated prion-infected mice differed considerably from untreated prion-infected mice (Fig. 5a). Analyses of expression signatures from CTEP-treated prion-infected mice compared to untreated RML mice at 90 dpi revealed that CTEP treatment resulted in both up- and down-regulated genes; however, only 28 genes were significantly differentially expressed (Fig. 5b, Supplementary Fig. S4a-c). Differentially expressed genes (DEGs) from prion-infected mice with and without CTEP treatment were analyzed for pathway enrichment. Interestingly, DEGs of CTEP-treated prion-infected mice did not form into any GO networks, thus, did not identify a specifically targeted pathway. However, several dysregulated genes including *CHAT*, *SEPT9*, and *GPD2* have been described to be involved in neurodegenerative diseases. When prion-infected mice (RML) were compared to uninfected control mice for DEGs, immune-related pathways stood out as the most dysregulated GO networks (Supplementary Fig. S5a-b). Of note, despite a significant prolongation of survival by CTEP treatment from 60 dpi onwards, neither immune-related pathways (Fig. 5b) nor microglial dysregulation (Supplementary Fig. S3a) was rescued by CTEP treatment.

### mGluR5 Inhibition by CTEP blocks Aβ- but not PrP^Sc^-mediated subacute synaptotoxicity in primary neurons

**Fig. 6:**
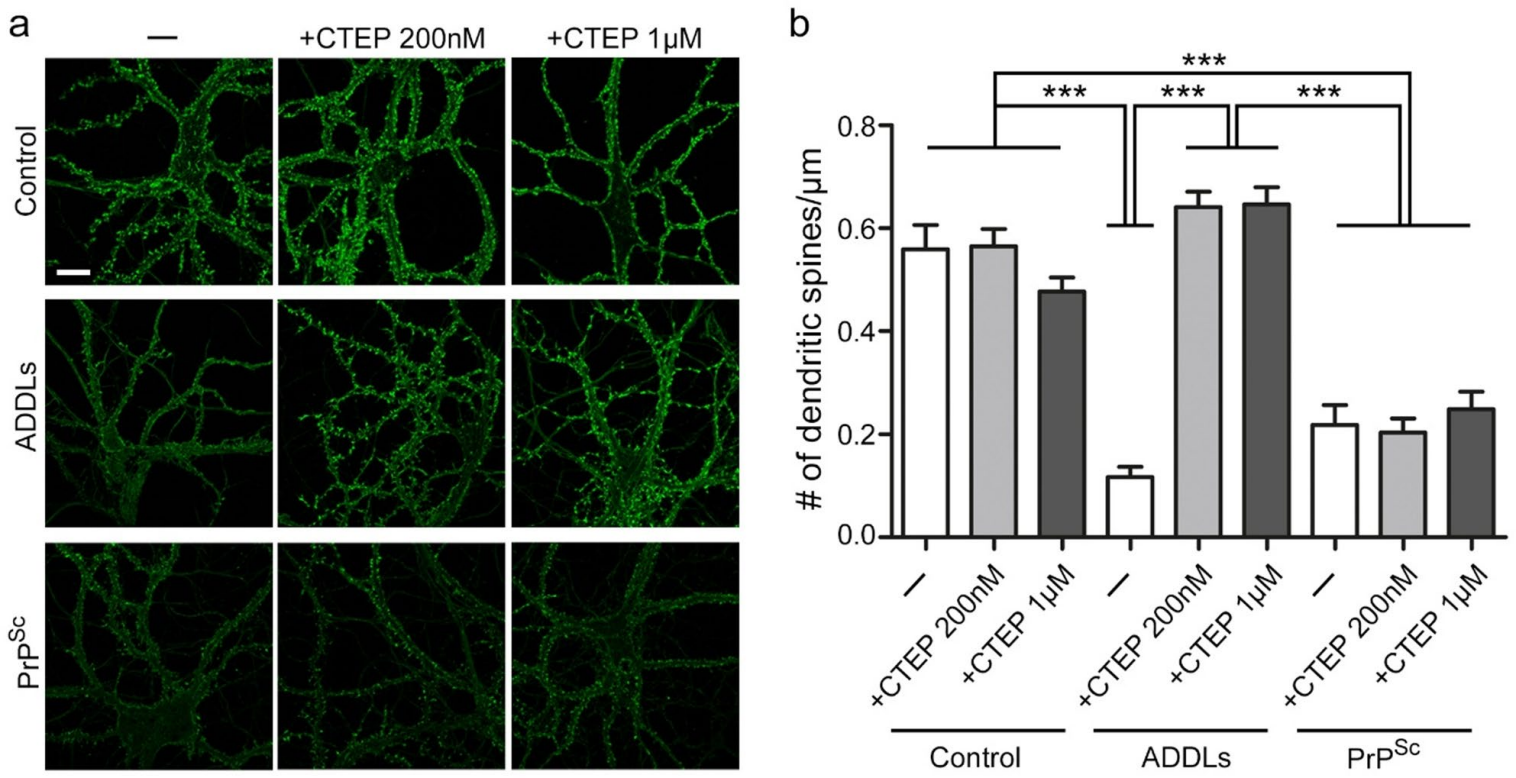
CTEP blocks subacute synaptotoxicity of Aβ but not PrP^Sc^ in primary neuronal cultures. **a** Representative images of primary hippocampal neurons cultured either with purified PrP^Sc^, with mock-purified material from uninfected brains, or with preparations of oligomeric Aβ (ADDLs) for 24 h in the presence or absence of 200 nM or 1 µM CTEP. Neurons were stained with Alexa488-phalloidin to visualize dendritic spines (green). Scale bar: 20 μm **b** Quantification of dendritic spine density upon Aβ or PrP^Sc^ exposure both significantly decreased the number of spines within 24 h. While treatment with CTEP significantly rescued synaptotoxicity of Aβ, it had no influence on loss of spine density associated with PrP^Sc^ (*n* = 12–15 cells per group from 3 independent experiments each; One-way ANOVA with Tukey’s multiple comparison test: ***p < 0.001)

To examine the impact of mGluR5 inhibition on synaptic integrity and neuronal health side-by-side in the context of prion or Alzheimer’s disease-associated pathology, we assessed the effect of CTEP on PrP^Sc^- and Aβ-induced synaptotoxicity in a primary neuron/astrocyte co-culture assay. We treated hippocampal neuron cultures (positioned over an astrocyte feeder layer) with purified PrP^Sc^, or with mock-purified material from uninfected brains, or with preparations of oligomeric Aβ (ADDLs) for 24 h in the presence or absence of CTEP (Fig. 6a). CTEP alone did not show an effect on dendritic spine density. As expected, treatment with either PrP^Sc^ or ADDLs led to a significant reduction in spine density (Fig. 6a, b). Remarkably, CTEP significantly blocked the synaptotoxic effect of ADDLs, but failed to prevent PrP^Sc^-induced spine loss (Fig. 6a, b).

### mGluR5 is dysregulated during the preclinical phase in prion diseases in different mammals

**Fig. 7:**
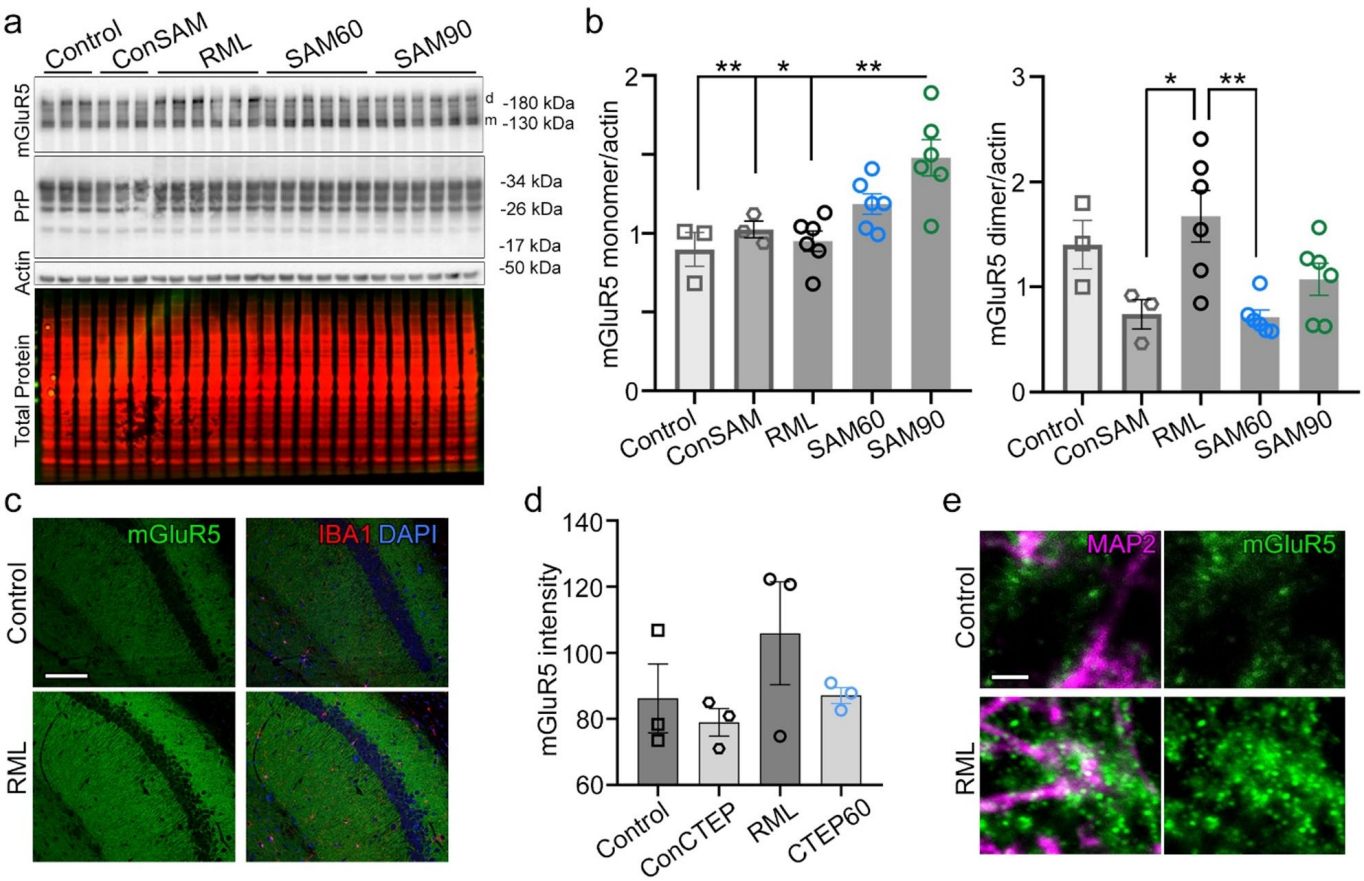
mGluR5 is dysregulated during the course of prion disease in mice. **a** Representative western blot of mGluR5, total PrP, and β-actin (Actin) from cortical tissues of SAM60, SAM90, RML, and control mice with (ConSAM) and without SAM treatment (Control) at 115 dpi (*n* = 3–6 per group). mGluR5 monomers are labeled with m, whereas mGluR5 dimers are labeled with d. Total protein staining served as a loading control. **b** Quantification of western blot signal of mGluR5 normalized to β-actin (actin) abundance shows a significant upregulation of dimeric mGluR5 in the untreated prion-infected RML mice that was absent upon SAM treatment. One-way ANOVA with Tukey’s multiple comparison test: Monomers **p = 0.0042; *p = 0.0293; **p = 0.0015, Dimers *p = 0.0285; **p = 0.0045. **c** Representative immunofluorescence staining of brain tissue from control and prion-infected RML mice at 90 dpi shows abundant expression of mGluR5 in the hippocampus region (mGluR5 (green), IBA1 (red), nuclei/DAPI (blue)). Scale bar: 90 μm. **d** Quantification of fluorescence signal showing a trend towards upregulated mGluR5 in the untreated prion-infected RML mice at 90 dpi. (*n* = 3 per group). **e** Expansion microscopy of hippocampus tissue (stratum radiatum of the CA1 region) from selected control and prion-infected RML mice at 90 dpi shows mGluR5 puncta (green) with increased size and signal intensity surrounding neuronal processes (MAP2, magenta) in prion-infected RML mice at 90 dpi. Scale bar: 0.8 μm

Recent findings suggest that mGluR5 protein abundance is dynamically dysregulated during prion disease, with early upregulation followed by later downregulation at clinical disease stages [46]. To explore this further, we analyzed mGluR5 abundance in cortex homogenates from prion-infected and control mice at 115 dpi, when first clinical symptoms are obvious such as stiff tail and gradual loss of nest building capabilities, with and without treatment with SAM using western blotting (Fig. 7a). Quantification revealed that the dimeric form of mGluR5 was significantly increased in prion-infected mice, whereas SAM treatment reduced this effect (Fig. 7b). Conversely, monomeric forms of mGluR5 levels were significantly elevated in SAM90 treated prion-infected mice when compared to prion-infected mice, but also in comparison to untreated mice (Fig. 7b). Immuno-histochemical detection of mGluR5 in adult wild type mice, show that mGluR5 is abundant throughout the entire brain (Supplementary Fig. S5a).

In the CTEP experiment, extra groups of mice were taken at preclinical time-point 90 dpi (30 days after treatment start on day 60). To assess whether abundance of mGluR5 is similarly dysregulated in this cohort and at this slightly earlier time point compared to the SAM mice (day 90 versus day 115 post infection) we investigated cortex brain homogenates by western blot analysis. Interestingly, in line with our data from the SAM experiment, mGluR5 was significantly upregulated in prion-infected mice (Supplementary Fig. S6b-c). However, also CTEP60-treated mice showed similarly high mGluR5 levels. Due to the sample age, it was not possible to discriminate dimeric and monomeric mGluR5. However, as a complementary method, abundance of mGluR5 was also assessed by immunofluorescence staining of brain sections (Fig. 7c). mGluR5 levels were highest in the hippocampus region, and quantification specifically in this region also revealed a trend towards increased mGluR5 signal in preclinical prion-infected mice (Fig. 7d). However, the differences were not significant, thus, highlighting the obstacles in quantifying protein levels of a rather diffuse protein staining. To compare mGluR5 levels at terminal disease stages, we stained brain sections at terminal prion disease and age matched control mice. In line with published data [46], mGluR5 was downregulated in the prion infected mice (Supplementary Fig. S6d). To more closely examine mGluR5 distribution in the hippocampus, we applied expansion microscopy to selected brain sections of prion-infected mice at day 90 post infection (RML) and age-matched control mice. Control mice showed the expected mGluR5 distribution in the hippocampus with small puncta surrounding neuronal processes. In contrast, in the brain of RML mice, mGluR5 signal presented with thicker dots of potentially clustered mGluR5 receptors (Fig. 7e).

**Fig. 8:**
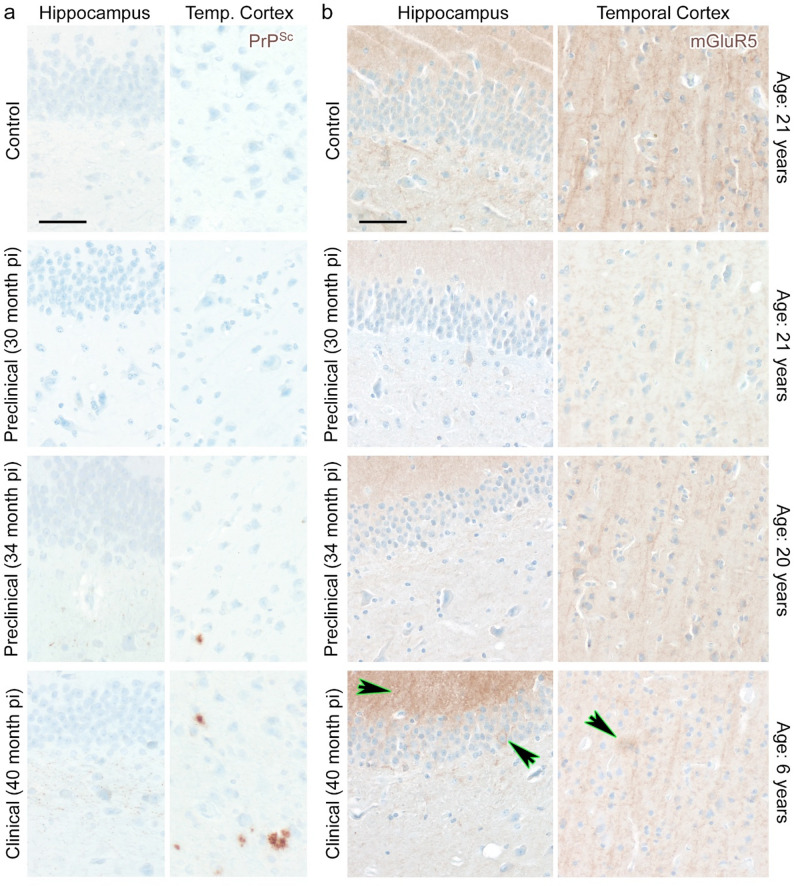
mGluR5 is dysregulated during the subclinical phase in a preclinical non-human primate model of prion disease. **a** Representative immunohistochemical staining of PrP^Sc^ in the brains of rhesus macaques is shown at given time points after intraperitoneal infection with human vCJD prions. Note the intra-neuronal thread-like PrP^Sc^ pattern in the hippocampus and the plaque-like PrP^Sc^ depositions in the temporal cortex in one of the preclinical and the clinical animal. Scale bar: 50 μm. **b** Representative IHC staining of mGluR5 in the hippocampus and temporal cortex of rhesus macaques infected with human vCJD prions. Of note, mGluR5 is reduced in preclinical prion disease at 30 months post infection when compared to a mock-infected control animal. At a late disease stage, mGluR5 abundance is still reduced in the temporal cortex, but is displaying a peri-neuronal and plaque-like staining pattern in both hippocampus and temporal cortex (green arrows). The ages of the individual rhesus macaques at the time of termination of the experiments in years are noted. Scale bar: 50 μm

Macaque monkeys represent highly valid disease models, since they show a very close to human brain gene expression [39]. To explore the abundance of mGluR5 in a non-human primate model of prion disease, we stained brain sections of macaque monkeys that were infected with human variant Creutzfeldt-Jakob disease (vCJD) prions. Due to intercurrent illness [28], some monkeys were sacrificed at time points at 30 and 34 months post infection, respectively, without showing signs of prion disease; and one animal progressed to clinical disease and was sacrificed at 40 months post infection. PrP^Sc^ deposition in brain tissue sections was assessed by immunohistochemistry and was detectable from 34 months post infection onwards in a typical plaque-like pattern, but also in intra-neuronal threads in the hippocampus (Fig. 8a). One animal at 30 months post infection did not show overt PrP^Sc^ deposits when analyzed by immunohistochemistry, but already had abundant PrP^Res^ in the cerebellum when detected by highly sensitive biochemical methods [28]. In the brain of one control monkey, the hippocampus and the cortex regions presented with strong mGluR5 abundance (Fig. 8b). In contrast, mGluR5 was severely reduced at both subclinical time points in the assessed brain regions (Fig. 8b). One animal with clinical prion disease presented with a mixed picture. While mGluR5 signal was reduced in the cortex, regions with patchy deposition could be detected in the cortex and single cells and neuronal processes showed very high amounts of mGluR5 in the hippocampus (Fig. 8b). Since it has been shown that mGluR5 abundance is reduced with aging in mice [15, 18, 20, 22, 54], we determined the age of the experimental animals at the time point of termination of the experiment (Fig. 8b). Interestingly, while the control animal and the prion-infected month 30 and 34 animals were all three rather old (>/= 17 years at experimental infection), the one animal that progressed to clinical prion disease was significantly younger (2.5 years at inoculation). Thus, the mixed mGluR5 presentation in this animal, with high abundance in some brain regions compared to the other three animals, but reduction and clustering of mGluR5 in other brain areas might well be attributed to the mix of young age and terminal prion disease (Fig. 8b).

Taken together, our data confirm that mGluR5 abundance is highly disturbed in different mammalian prion disease models, however, the underlying mechanisms and consequences will certainly need further exploration. We demonstrate here that mGluR5 inhibition in prion-infected mice can improve survival and behavior, but only when initiated as a prophylactic treatment, before onset of symptoms.

## Discussion

The effect of pharmacological or genetic inhibition of mGluR5 on disease pathophysiology has been shown in a variety of neurodegenerative diseases [15, 18, 20, 22, 54]. Our data show that chronic pharmacological inhibition of mGluR5 in a prion disease mouse model significantly prolonged survival, but only if treatment started before the onset of symptoms.

Surprisingly, inhibition of physiological glutamate signaling as well as misfolded protein signaling at mGluR5 by CTEP from an early preclinical time point onwards was superior to silent allosteric modulation (SAM). Thus, dysregulation of mGluR5 function might contribute to prion disease progression beyond its effect as a receptor of PrP-induced neurotoxic signaling. Silent allosteric modulation for mGluR5 by SAM was specifically developed in the context of AD to separate the mechanistic benefits of targeting PrP^C^-mediated neurotoxic signaling, which are addressed by SAM, from the complete inhibition of mGluR5 glutamatergic function. In contrast, CTEP affects and blocks both functions of mGluR5, glutamatergic signaling of mGluR5 and PrP^C^-mediated downstream pathways (the PrP^Sc^/PrP^C^/mGluR5 signaling axis). Thus, our data suggest that dysregulation of mGluR5 signaling in prion disease pathophysiology might affect both pathways and is distinct from that seen in AD.

Interestingly, prophylactic CTEP, in contrast to SAM treatment, significantly improved nest-building behavior. Although nest building has been used to assess behavioral changes and predict disease progression in prion-infected mice [9], interpreting these results in group-housed settings may not be robust in some comparisons. While individual behavioral assessments could overcome this limitation, such methods were not feasible in this study due to restrictions associated with working with prion-infected animals. Of note, prion-infected mice that were CTEP-treated from day 60 onwards also remained very agile and explorative in our study even during late disease phases. Moreover, in contrast to untreated prion-infected mice, they did not display the typical loss of grooming behavior. Unfortunately, we did not systematically document these observations. This is in line with the finding that the CTEP analogue Basimglurant was successful in treating symptoms of major depressive disorders in rat and mouse models [37]. Interestingly, evidence for mood alterations in the prodromal phase of the sporadic human prion disorder Creutzfeldt-Jakob disease has recently been noted [61]. Beneficial effects of mGluR5 inhibition on behavior have been described with both CTEP and SAM in mouse models for AD-related pathology [20–22]. Complete blockage of mGluR5 under normal physiological conditions might lead to unwanted side-effects. However, although knockout of mGluR5 has been shown to impair LTP in hippocampal CA1 but not CA3 in mice [38], such effects could also be derived from developmental dysfunctions due to the constitutive knockout of mGluR5 in those mice [3, 60]. In contrast, Hamilton et al. could not detect significant changes in behavior or memory in healthy control mice treated with CTEP [22].

Although treatment with CTEP reduced plaque formation and levels of soluble Aβ oligomers in the hippocampus and cortex of respective mouse models for AD-related amyloidosis [22], we could not detect major effects on PrP^Res^ levels in our prion mouse model. This is in line with former experiments showing unchanged PrP^Res^ levels using the mGluR-inhibitor MPEP over the entire experimental course in a prion mouse model [18]. However, deposition of PrP^Sc^ is already plateauing comparatively early during disease progression, independent of mGluR5, and does not necessarily correlate with the degree of neurotoxicity [52]. In contrast, spongiosis was attenuated by both drugs, however, to different degrees. The mechanisms underlying these regional differences between SAM and CTEP are not fully understood. Vacuolation profiles (spongiosis) in RML-challenged mice have been studied for decades, and it is well established that the RML prion strain induces more severe spongiosis in the thalamus than in the hippocampus and (neo)cortex [34]. Interestingly, more recent findings have implicated the protein PIKfyve, but also dysregulated protein degradation as driver of spongiosis [31]. Since CTEP is blocking both, the PrP^Sc^/PrP^C^/mGluR5 signaling axis and glutamatergic Ca2^+^ signaling which is dysregulated in prion diseases, its effect might well exceed that of SAM.

As a limitation of our study, both cohorts, the experiments with CTEP versus the experiments with SAM were not performed directly side-by-side, but consecutively. However, all relevant controls including prion-only infection and negative controls with and without treatment were included in both set-ups. From our experiences from the CTEP study, small adaptations were implemented in the SAM study. First, we used two slightly different dosages of RML prions for inoculation to refine our experiments following 3R strategies. However, RML-based mouse models have been shown to be highly reproducible in our and other hands [18, 29]. Second, we planned intermitted samples at day 115 post infection to include those mice that were treated from day 90 onwards. Interestingly, our data showed comparable results for both tested time points.

Mechanistic investigation in primary neuronal cultures revealed that, while PrP^Sc^-induced synaptotoxicity was not rescued, Aβ-mediated synaptotoxicity was effectively blocked by CTEP, highlighting a differential therapeutic potential depending on the nature of toxic misfolded protein oligomers and the timing of intervention. This fits data from others using primary neuronal or brain slice cultures revealing that blockage of glutamatergic signaling with MPEP in prion disease models act on the level of reducing neurotoxicity rather than synaptotoxicity [14, 18]. While oligomeric Aβ causes PrP^C^-dependent dendritic spine retraction in primary neurons via mGluR5 as binding partner [14], the different roles of the mGluR5 and PrP^C^ interplay in prion diseases are less well defined. Spine retraction might be a very early event in PrP^Sc^ neurotoxicity that may be mGluR5-independent, but other, mGluR5-dependent mechanisms may come into play during disease progression [14]. In prion diseases, PrP^C^ serves as a receptor for toxic β-sheet enriched PrP^Sc^ [6, 49, 50], but also as a substrate for the detrimental templated prion conversion cascade itself [48]. It is feasible that excessive PrP^Sc^ is interacting with alternative binding partners to induce neuronal distress and toxic signaling processes [16, 27, 35, 53]. Therefore, blockage of mGluR5 after this phase may be much less effective.

We noticed differences in the mRNA expression levels in our prion mouse model at 90 dpi when compared to non-infected control mice. Here, immune dysregulation associated pathways stand out. This is in line with published data that especially at this preclinical time point, the microglial signature is shifting [40, 41]. Global expression changes in the brain of RML prion-infected mice manifest between 60 and 90 dpi [26]. While we only assessed transcriptional signatures in the Hippocampus, region specific dysregulation of microglial profiles has been noted [40]. Surprisingly, treatment with CTEP only led to subtle changes in expression pattern in the hippocampus compared to non-treated prion-infected mice when assessed by bulk mRNA sequencing. These differences were not enriched in any pathway using our GO analysis and did not show amelioration of microglia-associated signatures as also shown by immunohistochemical analyses. Interestingly, we also did not see differences in PrP^Sc^ formation. Disconnection of PrP^Sc^ formation or misfolded protein seeding activity with microglial dysregulation in prion diseases have been noted before [2, 42]. Still, it is not entirely clear what drives microglia activation in prion diseases. In AD, we could specifically identify neuronal distress as driver of microglial signature switch [30]. However, transcriptional changes alone might not be able to show the full picture. Of note, mGluR5 is highly accessible to cytosolic binding partners and, upon complex formation with PrP^C^, can cause downstream activation of intracellular signaling cascades, including phosphorylation of kinases [32]. Accordingly, treatment with PrP^Sc^ in cultured neurons was shown to affect protein phosphorylation and let to phospho-p38-dependent downstream signaling cascades [14]. Moreover, mGluR5 dysfunction in prion diseases could be further fueled by mis-localization or clustering at the neuronal/synaptic membrane, since prion-mimetic antibodies were shown to increase mGluR5 clustering around dendritic spines, mimicking the toxicity of Aβ oligomers [18]. Using western blotting and expansion microscopy, we indeed observed increased dimerization and potential clustering of mGluR5 in the cortex and hippocampus of prion-infected mice.

Genetic ablation of mGluR5 led to reduced Aβ oligomers and plaques in APPswe/PS1ΔE9 mice [21]. In striking contrast, genetic knockout of mGluR5 did not improve survival in two independent prion disease mouse models [4, 18]. More recent data have shown that mGluR5 protein abundance is upregulated at early disease stages in a prion mouse model, but then significantly downregulated over the clinical disease course [46]. Interestingly, it was also shown that mGluR5 in mice is downregulated with aging in all investigated brain regions [4, 18]. Interestingly, by using immuno-histochemical detection of mGluR5 in adult wild type mice, we could show that mGluR5 is surprisingly abundant throughout the entire brain. We could also detect dysregulation of mGluR5 abundance in our prion disease mouse model and in non-human primates infected with human vCJD prions. However, the age-differences of the macaques in this study limited the interpretation of the clinical animal. Interestingly, increased mGluR5 protein levels contributed to cellular damage in prion-infected cells [25]. Given these and our data, we speculate that, in prion diseases, mGluR5 dysregulation might lead to malfunctions especially at early disease stages, thus, therapeutic manipulation of mGluR5 might no longer be effective at late disease stages. Interestingly, in a mouse model for fragile X syndrome, treatment with CTEP during a short preclinical time period was superior to chronic treatment, which was explained by timely inhibition of mGluR5 during a critical period [56].

Considering the differences in treatment effects in our experimental groups, we hypothesize that the critical therapeutic window for starting mGluR5-directed therapies in prion diseases is limited to the early preclinical period before or while mGluR5 is most dysregulated corresponding to 60–90 dpi in our study. In known cases of the rare human genetic prion diseases, the treatment with CTEP or analogues might be started before the onset of symptoms and could complement other more invasive therapeutic strategies [44, 45]. Although mGluR5 inhibition in our model significantly prolonged survival, the application of CTEP or other highly selective mGluR5 inhibitors for the treatment of sporadic human prion diseases might not be feasible. Unfortunately, for sporadic human prion diseases which are diagnosed rather late in the disease course, the narrow therapeutic window precludes their successful application.

## Supplementary Information

Below is the link to the electronic supplementary material.

Supplementary Material 1

## Data Availability

No datasets were generated or analysed during the current study.
