# Supplementary material for: Presymptomatic pharmacological inhibition of mGluR5 improves survival in a mouse model of prion diseases
Source: Acta Neuropathol Commun. 2026 Jan 29;14:83. doi: 10.1186/s40478-026-02235-9 (PMC13059540; doi:10.1186/s40478-026-02235-9)
Supplement: Supplementary file 1 — Supplementary Material 1 [file 40478_2026_2235_MOESM1_ESM.pdf]

Supplementary Figures

Presymptomatic pharmacological inhibition of mGluR5 improves survival in a mouse model of prion diseases

Yue Wang[1,a], Behnam Mohammadi[1,a], Christiane Hartmann[1,a], Kristin Hartmann[1], Edda Thies[1], Andreu Matamoros-Angles[1], Cheng Fang[2], David A. Harris[2], Jörg Tatzelt[3,4], Hermann C. Altmeppen[1], Diego Sepulveda-Falla[1], Stephen M. Strittmatter[5], Markus Glatzel[1], Susanne Krasemann[1]*

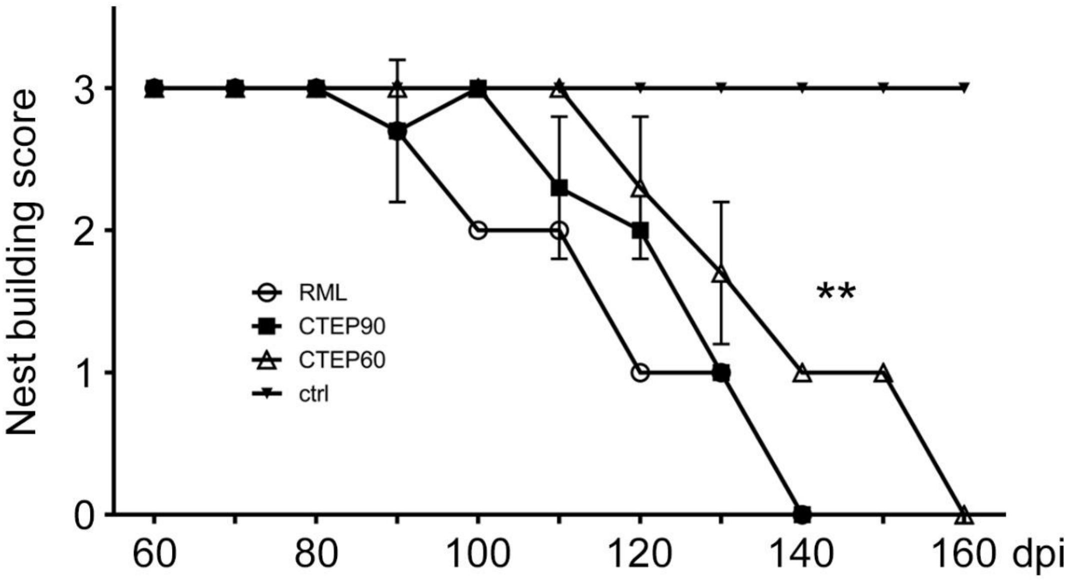

Supplementary Fig. 1 Preclinical treatment with the mGluR5-inhibitor CTEP improve nest building behavior in prion-infected mice

Nest building was assessed blinded to the experimenter and scored on a scale from 0 to 3, where 3 was a tidy and perfect nest and 0 was no nest at all with all material stuck to the ground (see methods for detail). Semi-quantitative assessment of nest-building behavior in prion infected mice without (RML; n=9) and with CTEP treatment from day 60 (CTEP60; n=9) or day 90 (CTEP90; n=9) dpi onwards. Mice treated with CTEP from day 60 pi onwards showed a significant improvement of nest building behavior. One-way ANOVA with Brown-Forsythe multiple comparison test: p= 0.0072.

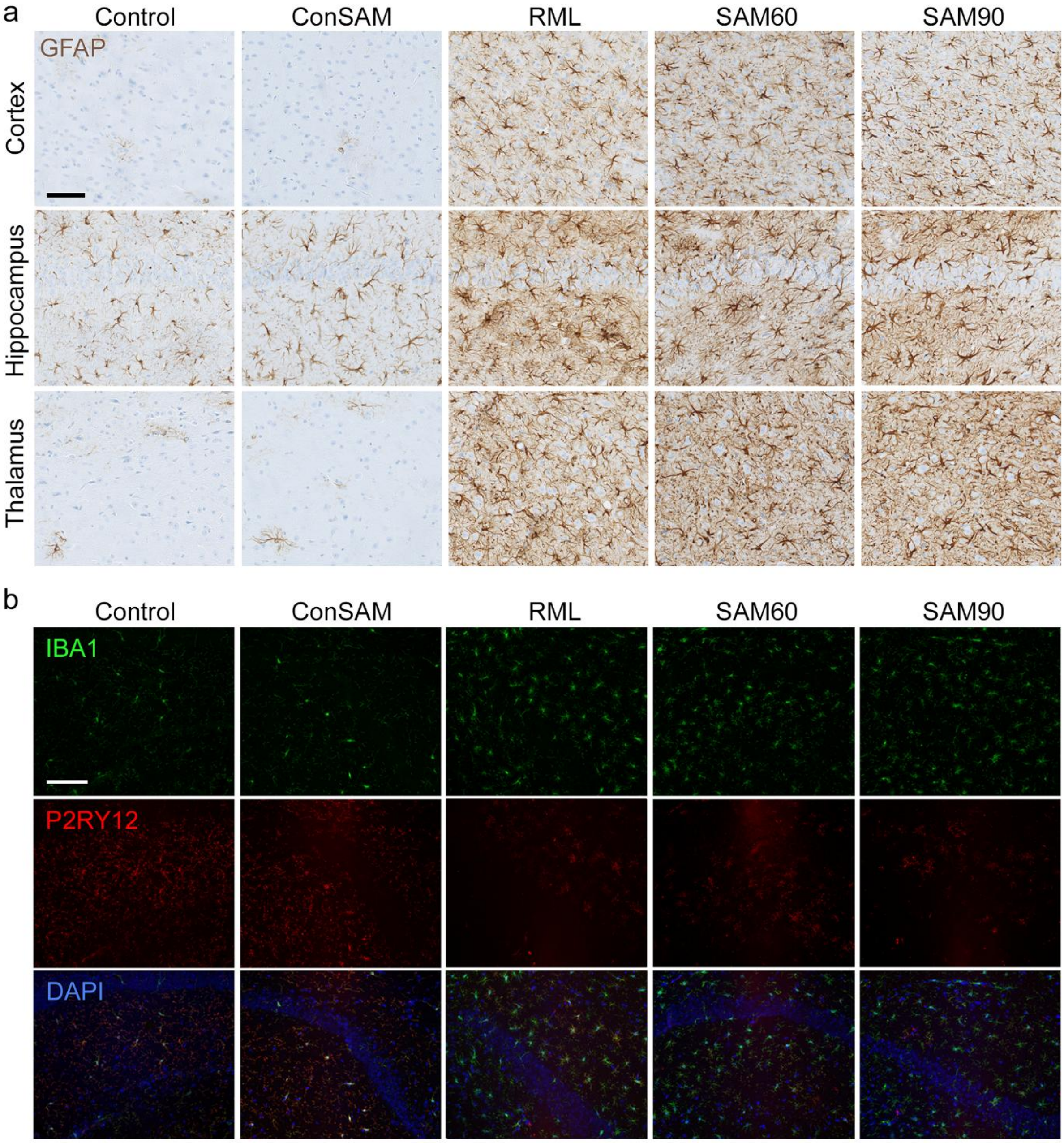

Supplementary Fig. 2 Unchanged levels of Microglia and Astrocyte activation upon SAM treatments

(a) Representative immunohistochemical staining for astrocytes (GFAP) in different brain regions at day 115 pi (RML; SAM60; SAM90) and healthy control mice with and without SAM treatment (Con; ConSAM) showed no difference in dependence of SAM treatment. As expected, severe changes in the morphology or abundance of astrocytes in prion infected mice could be detected that were unaffected by SAM treatment. Scale bar: 60µm. (b) Representative immunofluorescence staining for microglia (IBA1, green) and the microglial

homeostasis marker P2RY12 (red) in different brain regions at day 115 pi (RML; SAM60; SAM90) and healthy control mice with and without SAM treatment (Con; ConSAM) showed no difference in dependence of SAM treatment. However, severe changes in the morphology or abundance of microglia cells with downregulation of P2RY12 in prion infected mice could be detected that were unaffected by SAM treatment. Nuclei/DAPI (blue). Scale bar: 90µm.

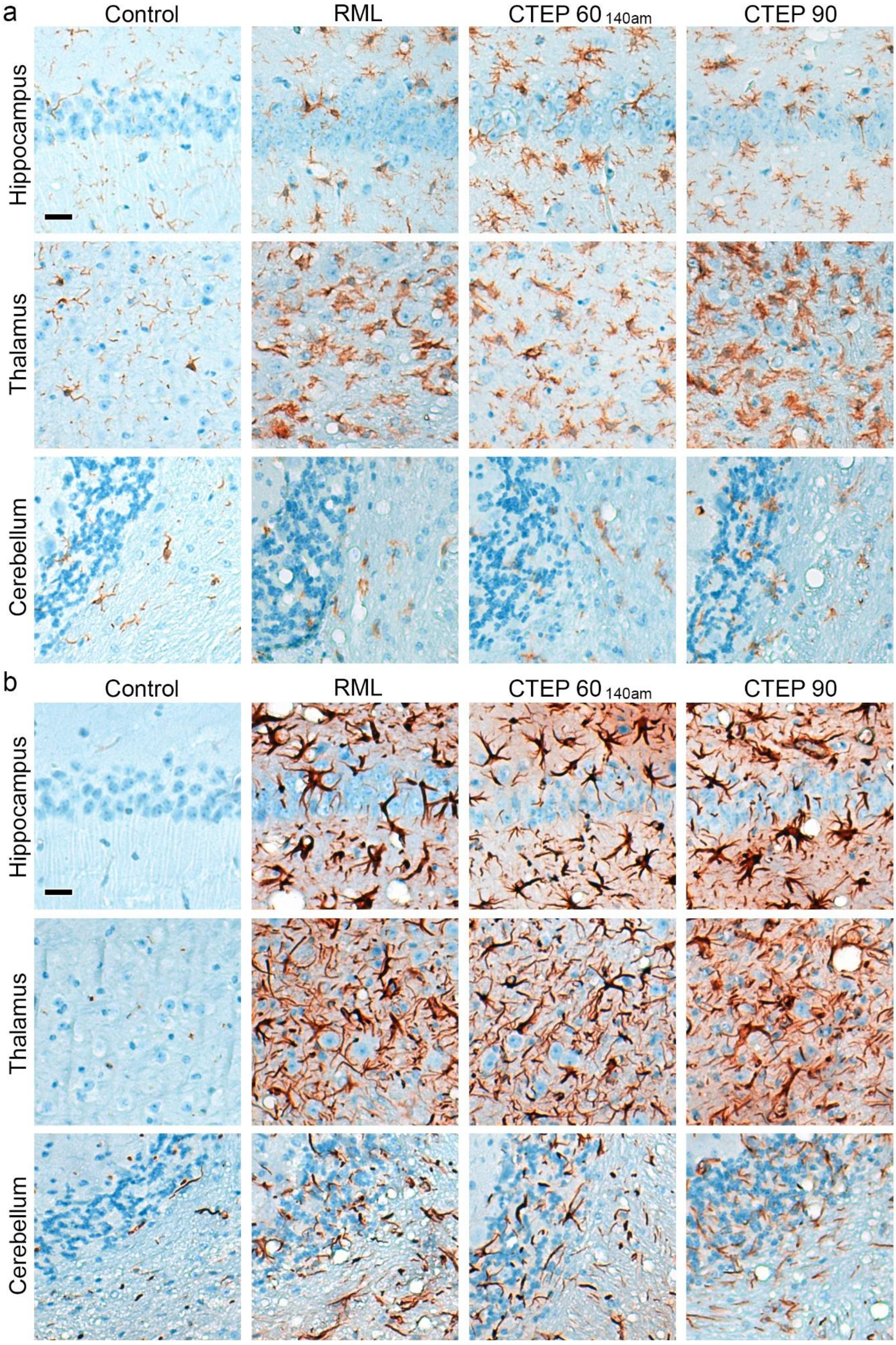

a
Control
RML
CTEP 60 140am
CTEP 90
Hippocampus
Thalamus
Cerebellum
b
Control
RML
CTEP 60 140am
CTEP 90
Hippocampus
Thalamus
Cerebellum

Supplementary Fig. 3 Unchanged levels of microglia and astrocyte activation upon CTEP treatments

Representative immunohistochemical staining for (a) microglia (IBA1) or (b) astrocytes (GFAP) in hippocampus, thalamus, and cerebellum in terminally diseased mice (RML; CTEP90), healthy control mice (Control), or age matched mice CTEP60 taken at 140 dpi ($CTEP60_{140am}$) showed no difference in the morphology or abundance of microglia cells or astrocytes in dependence of CTEP treatment compared to untreated prion infected mice. Scale bars: 20µm.

a

Differential Expression Counts:

	Up	Flat	Down
**Con – RML**	4	55368	47
**Con – ConCTEP**	2	55416	1
**Con - CTEP**	14	55272	133
**RML – ConCTEP**	77	55340	2
**RML – CTEP**	3	55391	25
**ConCTEP - CTEP**	10	55249	160

b

Up-regulated
GPD2
GJB4
CHAT

c

Down-regulated
DQ702453
BC005561
AK018302
1810022C23Rik
LAMA5
OLFR570
HSD11B1
SIX3OS1
RAD54L
GM15348
TCF21
CCDC114
KIF12
TJP3
1110038D17Rik
GSC
GPIHBP1
D430042O09Rik
SEPT9
AK158196
LRRC46
CPN1
SIX3OS1
FHAD1
TMEM87B

Supplementary Fig. 4 Treatment with CTEP leads to mild changes in the expression signature in hippocampus

Transcriptome analysis of hippocampus tissue samples of mice at 90 dpi with or without treatment with CTEP for 30 days is shown. (a) The table summarizes the differentially

expressed genes (DEGs) between the different group comparisons. Control mice show almost no changes upon CTEP treatment. (B) List of DEGs between untreated (RML, n=4) and treated (CTEP, n=4) prion infected mice that are up-regulated. (c) List of DEGs between untreated (RML, n=4) and treated (CTEP, n=4) prion infected mice that are down-regulated.

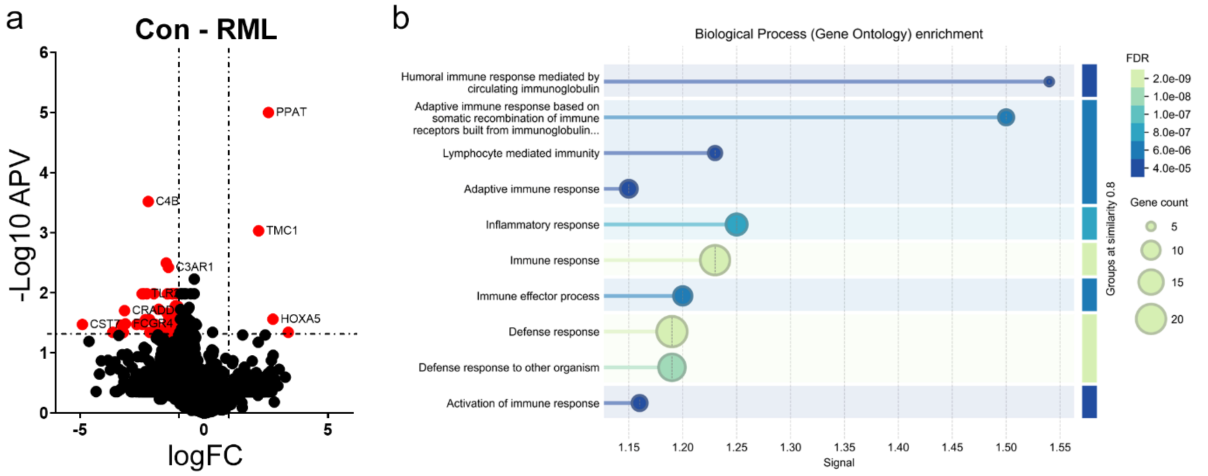

Supplementary Fig. 5 Immune pathways are dysregulated in the hippocampus in subclinical prion disease

(a) Volcano plot of the DEGs of ~~CTEP-treated~~ control mice (Con~~CTEP~~) (n=4) versus ~~untreated control mice~~ (n=4) ~~showed that only very few genes are affected by CTEP treatment in healthy mice.~~ (b) Gene Ontology enrichment analysis of DEGs from prion-infected mice (RML) (n=4) versus healthy control mice (n=4) at 90 dpi showed that mainly immune activation processes stand out at this subclinical time point.

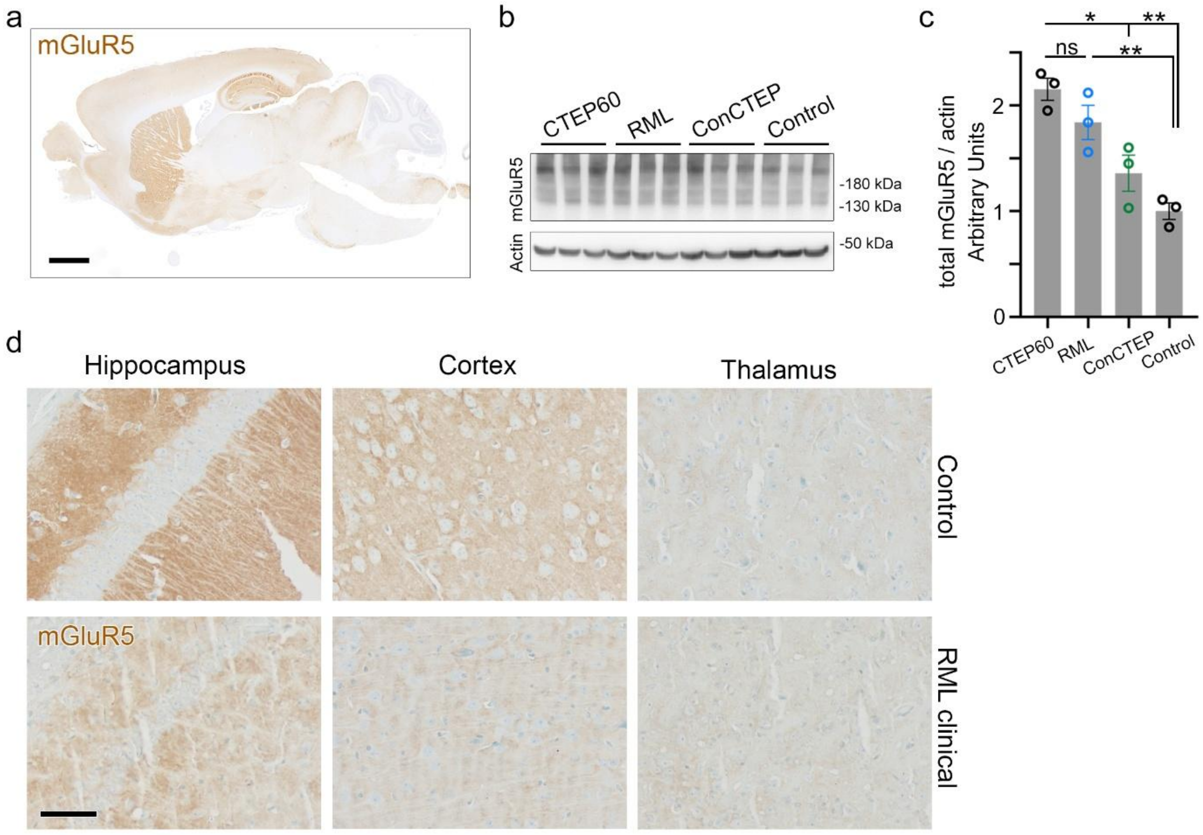

Supplementary Fig. 6 mGluR5 is upregulated at day 90 in prion infected mice

(a) Representative staining of mGluR5 in the brain of a healthy mouse displays that mGluR5 is highly abundant in almost all brain regions. Scale bar: 1mm. (b) Representative western blot of mGluR5 in cortex tissue at day 90 showed that mGluR5 is upregulated upon prion infection. (c) Quantification of western blot signal of mGluR5 normalized to β-actin (actin) abundance shows a significant upregulation of mGluR5 in the untreated prion-infected RML mice that was similar upon CTEP treatment. One-way ANOVA with Tukey's multiple comparison test. (d) Representative staining of mGluR5 in the brain of a terminally prion diseased mouse in comparison to an age matched animal showed the downregulation of mGluR5 in several prion disease-relevant brain areas. Scale bar: 50µm.

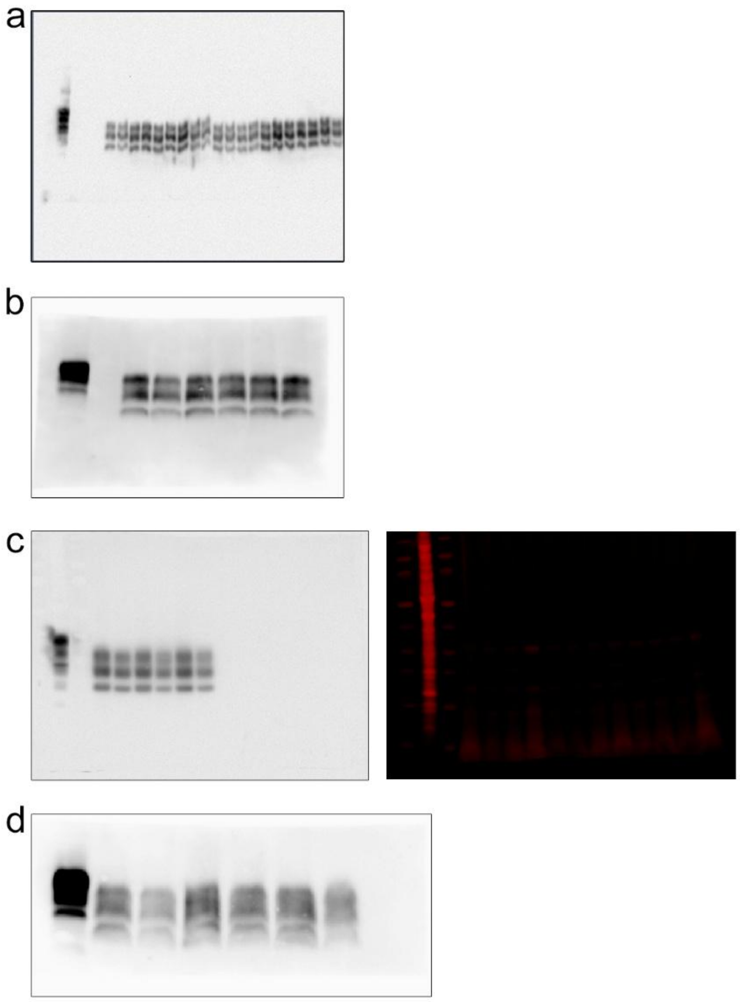

Supplementary Fig. 7 Uncropped western blots of PrP^{Res} relating to Figure 2a and Figure 4

(a) Uncropped western blot related to Figure 2a. Brain sample homogenates that were run on the same gel, but unrelated to this project, were cut from the right side of the blot. (b-d) Uncropped western blot related to Figure 4. (b) Representative western blot of PrP^{Res} at terminal and age-matched time points after CTEP treatment. (c) Representative western blot of PrP^{Res} in cortex at day 90 after CTEP treatment including total protein staining to proof the full digestion of the samples including the negative samples. Only the non-digested control is still detectable for total protein amount. (d) Representative western blot of PrP^{Res} [illegible]allamus at day 90 after CTEP treatment. Note that another type of polyacrylamide gel was used.

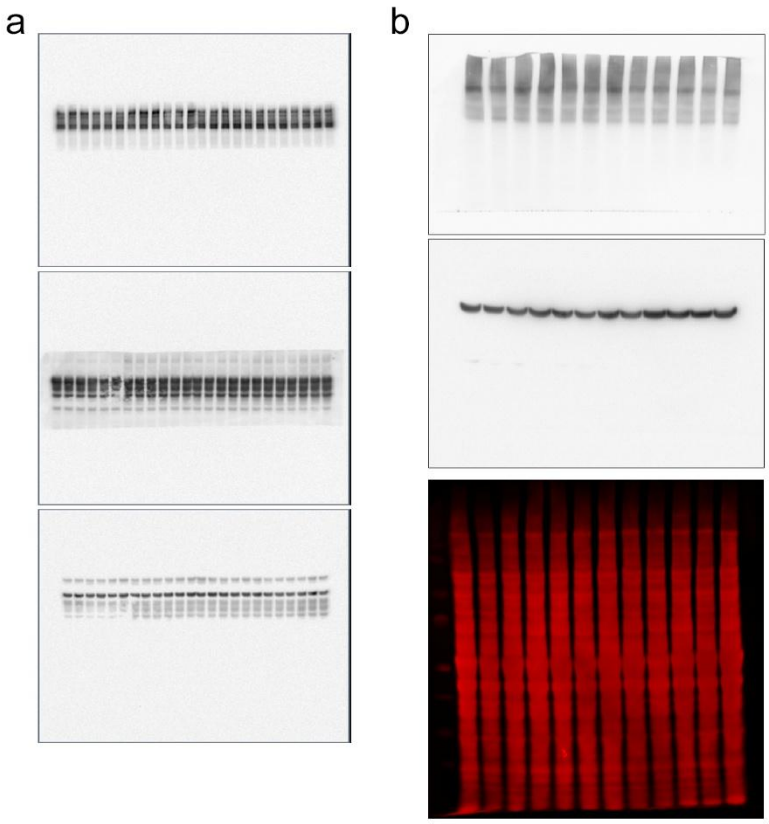

Supplementary Fig. 8 Uncropped western blots relating to Figure 7 and Supplementary Figure S6

(a) Uncropped western blot related to Figure 6a (SAM-treatment day 115). The membrane was cut in half after blotting and the upper part was incubated with the anti-mGluR5 antibody. The lower part of the membrane was first detected with an antibody against PrP. After stripping, this membrane part was re-probed with an antibody against actin. Despite stripping, residual signal from the PrP detection is still visible in the lower part, however, not overlapping with the signal from actin. (b) Uncropped western blot related to Supplementary Figure S6 (CTEP-treatment day 90). The membrane was cut in half after blotting and the upper part was incubated with the anti-mGluR5 antibody. The lower part of the membrane was detected with an antibody against actin.
